# Physical activity, sleep pattern and problematic internet use in undergraduate health-sciences students of Nepal – A cross-sectional study

**DOI:** 10.1371/journal.pdig.0000797

**Published:** 2026-07-23

**Authors:** Jay Prakash Jha, Hiramani Prasad Chaudhary, Dilli Bahadur Pun, Lok Raj Joshi, Kapil Amgain

**Affiliations:** 1 Department of Physiology, Pokhara Academy of Health Sciences, Pokhara, Gandaki Province, Nepal; 2 Department of Physiology, Nepalganj Medical College and Teaching Hopsital, Banke, Lumbini Province, Nepal; 3 Department of Clinical Physiology, Karnali Academy of Health Sciences, Jumla, Karnali, Nepal; 4 Department of Physiology, Faculty of Health Sciences, Far Western University, Dadeldhura, Far Western Province, Nepal; 5 Department of Clinical Anatomy and Cell Biology, Karnali Academy of Health Sciences, Jumla, Karnali, Nepal; Jos University Teaching Hospital, NIGERIA

## Abstract

Unregulated internet use may contribute to problematic internet use (PIU), which impairs health in physical, emotional, social, and functional domains. Excessive use of digital media also bears the potential to affect sleep and physical activity levels, which in turn adversely affect overall well-being. However, evidence on these issues in Nepal is limited. This study investigated patterns of digital device use, sleep, and physical activity among undergraduate health-sciences students in Nepal, and examined the frequency and predictors of PIU. A cross-sectional online survey was conducted among 362 students (206 females; median age 21 years) during January to August 2023 using a semi-structured questionnaire. About a third students (30.45%) reported inadequate sleep and a similar number (33.43%) were physically inactive, with females being more inactive (47.57%) than males (14.74%). PIU was highly prevalent (57%) and was significantly associated with poor sleep quality, low physical activity, and prolonged device use. Multivariable analysis confirmed that sex, sleep quality, physical activity, and computer knowledge were independent predictors of PIU. These findings highlight that poor sleep, low activity and PIU are common and interrelated in Nepali health-sciences students. Addressing these behaviours is important for safeguarding the students’ academic performance, mental health, and long-term well-being. Future research with a prospective study design including diverse sample could provide further insights into the complex relationship between PIU and its predictors.

## Author summary

This study addresses the relationship between digital technology use and the well-being of students. We examined patterns of physical activity, sleep, and internet use, focusing on problematic internet use (PIU) among undergraduate health-sciences students of Nepal. Our findings reveal that high digital device usage and PIU, low physical activity, and sleep problems were common in this population. These findings highlight the need to encourage healthier lifestyle choices in the era of digital academic fields. Our main goal is to raise awareness of excessive screen use and promote balanced habits. Finally, it encourages other researchers to explore further on the causal relation between lifestyle parameters and screen use, while contributing to awareness of healthy digital life as an emerging public health concern.

## 1. Introduction

College students are at their significant stage of life where they are likely to have poor health practices. Sedentary behaviour, encompassing activities such as internet browsing, phone communication, and social media interaction, potentially affects their sleep and physical activity level, which in turn has multiple detrimental health effects such as metabolic syndrome, cardiovascular diseases, cognitive decline, and all-cause-mortality [1,2].

The global proliferation of digital devices and internet connectivity underscores the escalating reliance on digital technology. As of October 2025, 73.2% of the global population enjoys internet access, with smartphones serving as the primary conduit for 70.1% of total population. This is the rise of 5.1% internet users year-over-year [3]. The daily internet use averages to six and half hours. Nepal is also seeing a growing internet penetration rate of 51.6% in 2023, which is rising (currently 55.8% in mid-2025). Social media engagement emerges as a dominant online activity in Nepal, where people spend an average of over 2 hours daily, predominantly via mobile devices [4].

This national trend extends to young students, including medical undergraduates, many of whom live independently in hostels, where unsupervised digital use may contribute to a higher propensity for internet dependence [5]. Research indicates that internet dependence, akin to substance abuse or compulsive eating, can be objectively evaluated and manifests in various physical and mental health complications [6].

Dependency on digital gadgets causes various physical and mental issues [7]. Excessive and unhealthy use of internet has been studied for its effect in different dimensions of health, impacting academic performance and future career prospects. Problematic Internet Use (PIU) is the condition characterised by excessive, impulsive, or poorly regulated engagement to internet that disrupts physical, emotional, social, and functional aspects of health [8]. It reflects a range of behavioural and psychological patterns such as compulsive use, neglect of duties, diminished social interaction, and loss of control, that are particularly disruptive in student populations, where academic performance and psychosocial development are at stake. PIU is conceptually related to internet addiction, though it represents a broader and often less severe spectrum of maladaptive internet behaviours [8]. Evidence of this disruption is reflected in a study by Balhara et al in 2019, which included 2634 participants from 8 countries, and showed that Nepal had the highest prevalence of PIU (12.6%) as per the Generalized Problematic Internet Use Scale-2 (GPIUS2) scale [9]. In contrast, Sharma *et al.* reported in 2018 a 32% prevalence among Nepali medical students using the same scale [10]. Another study done in college students of Chitwan and Kathmandu found the prevalence of internet addiction to be 35%, and that it mediated the effects of sleep quality on depressive symptoms [11].

Study also underscores the correlation between periods of heightened academic demand, inadequate sleep, and diminished academic achievement among university students [12]. Given the growing recognition of digitally mediated behaviours as key determinants of health, comprehensive evaluation and monitoring of daily health-related practices are essential for the formulation of targeted guidelines tailored to the students. Despite the critical need, there remains a paucity of research in Nepalese population concerning this multifaceted issue. These concerns fall within the broader scope of digital health—particularly where they intersect with mental wellbeing, behavioural regulation, and public health disparities.

Based on the known impacts of digital device use on health-related behaviours, we hypothesised that problematic internet use would be prevalent among health-sciences undergraduates in Nepal and would be associated with reduced sleep quality and lower physical activity.

### 1.1. Objective

The study aims to examine the patterns of computer gadget (screen) use, sleep habits, and physical activity, and to explore the association of PIU with computer use, sleep pattern and physical activity level among undergraduate health-sciences students of Nepal. This study finding will contribute to the understanding of technology-related behavioural health within a digital public health framework.

## 2. Methods

### 2.1. Ethics statement

Ethical clearance was taken from Ethical Review Board of Nepal Health Research Council (Reg no. 499/2022 P, date 5 January 2023) before starting the study. Written informed consent were taken from each of the participants before collecting data.

### 2.2. Sample

A cross-sectional study was conducted on the undergraduate health-sciences students of different colleges of Nepal. There are total seven provinces in Nepal, out of which, one (province no. 7) had no medical college at the time of investigation. We chose one medical college purposively from each of the six provinces. In each college, participants were enrolled by snowball method, including all the programs present in the institute. Target population of this study was undergraduate health-sciences students enrolled in MBBS, BDS, nursing, and paramedical sciences (public health and pharmacy). The population size was 4697 including all the undergraduate programs enrolled in year 2023 AD [13]. All the consenting undergraduate students of different programs of health-science study were included in the study. Students with diagnosed sleep disorder or physical disability were excluded. Calculated minimum sample (using the formula N = z^2^pq/d^2^) was 333, taking p (prevalence of problematic internet use) as 31.9% [10] with a 95% confidence interval and 5% margin of error. Accounting for 10% attrition rate, we aimed for data collection from 370 students and finally, we were able include 362 students. Although the final sample size was slightly below the target due to participant exclusion and non-response, it remained close to the estimated requirement and was considered adequate for the aim of prevalence estimation. Analyses of associations were exploratory, and the study was not powered to detect specific effect sizes.

### 2.3. Questionnaire

Semi-structured questionnaire was prepared by literature review, and included the different habits of students within past 15 days. It consisted of following parts: (a) general demography, including age, sex, course, address, phone number and email address; (b) sleep time and sleep disturbances (modified from the Pittsburgh Sleep Quality Index) [14]; (c) Physical activity as per International Physical Activity Questionnaire (IPAQ) [15]; (d) digital device usage habits (types of device used, duration of use of the device and internet, common uses of the gadgets, knowledge of computer technology, and usefulness of internet in their study); and (e) Problematic Internet Use (PIU) Questionnaire developed from former PIUQ [16]. Sleep habits included average duration of sleep, sleep latency, and wake up frequency during night. For scoring purpose, the normal sleep habit parameters were classified as 0 for normal and 1 for abnormal as per National Sleep Foundation (NSF) guideline [17] and another study by Hirshkowitz et al. [18]. Sleep disturbances were asked as frequency in 3-point likert scale and scored as 0 for never or rarely, 1 for occasional and 2 for frequently or always. Sleep disturbances included conditions such as trouble falling asleep, thoughts racing at sleep onset, frequent interruption of sleep, difficulty returning to sleep after an interruption, early morning waking, snoring, and excessive daytime sleepiness. Total sleep score ranged from 0 to 17 (1x3 for each abnormal scores in sleep habits, plus 7x2 for maximum frequency of sleep disturbances), higher score indicating poorer overall sleep quality.

Physical activity level was assessed for the types and intensity (heavy exercise, moderate exercise and walking) they performed within past two weeks. Following formula was used to calculate the weekly MET value as per the activity (According to IPAQ interpretation protocol [15]):

MET by heavy exercise: minutes of exercise per day x 8 x number of days per week
MET by moderate exercise: minutes of exercise per day x 4 x number of days per week
MET by walking: minutes of walking per day x 3.3 x number of days per week

Total MET value for each participant were counted by adding up the MET values by different activities. Physical activity level was classified according to IPAQ protocol as inactive, minimally active and Health Enhancing Physical Activity (HEPA) level active.

The Problematic Internet Use Questionnaire (PIUQ) included 14 questions about the frequency of the problems related to excessive internet use such as “How often does your internet use impair your work or study?” and “How often do you want to decrease internet usage, but you cannot succeed?”, with 5-point Likert scale response from 1 (for never) to 5 (for always). The score ranged from 14 to 70; higher score indicating more problem. To diagnose PIU in our study, the scale’s median score 42 was chosen as cut-off point; participant scoring 42 or above was labelled as PIU positive. The questionnaire was modified from Demetrovics (2008) [19] based on the context of Nepalese students, and has been utilised in a local population of Nepal as well [20]. They were later reviewed by experts as well and refined accordingly.

The questionnaire were built in jotform for distribution - a browser-based web form that could be used in any device and submitted online. Students were approached via social media or phone calls, and the link to the form was sent to the approachable students as per personal communication, who distributed it to the peers by snowball method. The initially approached student ensured the link did not leak outside the college. The CHERRIES guideline was followed for online survey [21]. No personally identifiable data were collected, but multiple submissions from any single user was prevented by automatic IP address analysis. The platform also used javascript to alert respondents on incomplete submission. Subjects with more than 10% of incomplete columns were planned to be rejected.

All the relevant information, the procedures of the study and rights of the participants were explained to each in person, via online forms or in phone before recruiting them. Participants’ consent was collected by online form which was to be submitted separately at the time of filling the form. Data were collected from January to August 2023.

### 2.4. Covariates

Quantitative variables included: age, annual family income (in NPR), bedtime and wake-up time, sleep duration (hours), sleep latency (minutes taken for falling asleep from the bedtime), sleep score, number of MET-minute per week, category of physical activity, duration of internet use (years), daily hours of screen use and internet use, and PIU score.

Qualitative variables were gender; sleep problems; different uses of gadgets; different social media platforms; and knowledge, interest in and usefulness of gadgets.

### 2.5. Statistical analysis

Descriptive statistics (frequency, percentage, mean, standard deviation, median, interquartile range) were used to summarise the data. Group comparisons were performed using Chi-square tests for categorical variables and Wilcoxon Rank-sum tests for continuous variables with non-normal distribution. Associations between continuous variables were examined using Spearman’s correlation. Linear regression analyses were conducted to explore predictors of problematic internet use. All analyses were performed using SPSS version 22, with statistical significance set at p < 0.05.

## 3. Results

Total 362 students participated in the study, of which 156 (43%) were males. Their mean age was 22 (±2.34 SD), ranging from 19 to 32 years. Students belonged to wide variety of location, from Morang in east to Bajhang in west of Nepal. Participants’ self-reported substance abuse habit was low: 10 reported as smoker (2.76%), 54 were occasional drinkers (14.92%), and one was heavy drinker (0.28%). Four participants did not respond to the substance abuse question. The average income of 279 respondents’ family was NPR 8,44,169 ± 17,51,658 per annum. Age and annual income was not significantly different between genders (Table 1).

**Table 1:** Frequency of different parameters in the participants and their gender-wise comparison.

Parameters	Median Total (IQR)	Median (IQR) Male	Median (IQR) Female	P value (Wilcoxon Rank-sum test)	Effect Size
Age (years)	21 (20 – 23)	22 (21 – 23)	21 (20 – 23)	0.398	0.00
Annual Income (x 100,000 NPR)	5 (2 – 10)	5 (2 – 8)	6 (1.5 – 12)	0.186	0.01
**‍Sleep Parameters**					
Bed time	12 am (11 pm – 12 am)	12 am (11:30 pm – 12:30 am)	11 pm (10 pm – 12 am)	<0.001*	0.04
Wake up time (am)	7 (6:30 – 7:30)	7 (6:30 – 7:30)	7 (6:30 – 7:30)	0.118	0.01
Sleep duration (hours)	7 (6 – 8)	7 (6 – 7)	7 (6.5 - 8)	0.001*	0.03
Sleep latency (minutes)	15 (10 – 30)	15 (10 – 30)	15 (10 – 30)	0.297	0.00
Wake up frequency	0.5 (0 – 1)	0 (0 – 1)	1 (1 – 2)	0.02*	0.02
Sleep Score	4 (3 – 6)	4 (3 – 6)	4 (3 – 6)	0.388	0.00
**‍Physical Activity**					
MET-minute per week	942 (459-1572)	1389.5 (901.125 – 2478.75)	652 (284.25 - 1110.25)	<0.001*	0.20
**Device and internet use**					
Internet use (years)	7 (5 – 8)	7 (5 – 8)	7 (5 – 8)	0.800	2.092e-4
Screen use weekdays (hours)	4 (3 – 5)	4 (3 – 5)	4 (3 – 5)	0.067	0.01
Screen use weekend (hours)	6 (5 – 8)	6 (4.5 – 8)	6 (5 – 8)	0.473	0.00
Internet use weekdays (hours)	3 (2 – 5)	3 (2 – 4)	4 (2 – 5)	0.010*	0.02
Internet use weekend (hours)	5 (4 – 7)	5 (4 – 7)	6 (4 – 8)	0.025*	0.01
PIU score	43 (37 – 50)	43 (35 – 50)	43 (37 – 50)	0.565	9.406e-4

* Statistically significant at 95% confidence.

### 3.1. Sleep

The median bed time for the students was 12 midnight and wake up time was 7 am, making seven hours as their median sleep duration (Table 1). Four students did not respond to the sleep habit questions. Out of 358 respondents, about a third (109, 30.45%) got sleep for less than 7 hours, and 4 (1.12%) had more than 9 hours, which are abnormal for adults as per National Sleep Foundation [17]. Only 245 (68.44%) students had adequate sleep duration of 7–9 hours. Nineteen (5.31%) students had prolonged sleep latency of more than 30 minutes (maximum latency 2 hours) (Table 2).

**Table 2:** Sleep habits and disturbances in the participants (according to habit in past 2 weeks).

Regular sleep habit			
Parameters (n = 358)		frequency	Percent
Sleep duration (hours) (Range 4–12 hours)	7 to 9 (normal)	245	68.44%
	<7 (inadequate)	109	30.45%
	>9 (excessive)	4	1.12%
Sleep latency (minute) (Range 0–120 minutes)	<30 (normal)	339	94.69%
	>30 (prolonged)	19	5.31%
Wake up frequency at night (Range 0–7)	0 to 1 (normal)	292	81.56%
	>1 (frequent)	66	18.44%
**Sleep Disturbance**			
Sleep disturbances (n = 360)	Never or rarely	Occasionally	Frequently
Trouble falling asleep	159 (44.17%)	168 (46.67%)	33 (9.17%)
Thoughts racing too much while in bed	100 (27.78%)	179 (49.72%)	81 (22.50%)
Frequent waking during sleep	266 (73.89%)	79 (21.94%)	15 (4.17%)
Difficulty returning to Sleep	194 (53.89%)	134 (37.27%)	32 (8.89%)
Early morning waking	128 (35.56%)	147 (40.83%)	85 (23.61%)
Snoring	291 (80.83%)	57 (15.83%)	12 (3.33%)
Excessive daytime sleepiness	111 (30.83%)	189 (52.50%)	60 (16.67%)

Among the 360 students responding to sleep disturbances, early morning waking was the most common disturbance reported to be frequent (23.61%), followed by thoughts racing too much in bed (22.5%). Snoring was frequently experienced by 12 students (3.33%).

Among the 356 participants who answered completely on the sleep habit section, the median sleep score in our sample was 4. Although males’ sleep time (median time 12 am vs 11 pm) was significantly more delayed and had lower sleep duration (IQR 6 – 7 vs 6.5 – 8) than females’ and females woke up more frequently than males during their sleep (median 1 vs 0), there was no significant difference in total sleep score between them (p = 0.388, Table 1).

### 3.2. Physical activity

All students responded to the physical activity question. Based on the self-reported form, most of them did not engage in heavy exercise. Total mean MET value was 1323.19 ± 1385.32 MET-min/week and median was 942 (IQR 459–1572) MET-min/week. Comparison between gender shows that males had significantly more MET value than females (p < 0.001, Table 1). More than half of the participants were minimally active (55.8%), and one third were inactive (33.43%). Males were significantly more active than females (Chi square 57.6, p < 0.001) (Table 3).

**Table 3:** Physical activity level in male and female students.

	Inactive	Minimally active	HEPA active	Chi Square	P value
**Male**	23 (14.74%)	100 (64.1%)	33 (21.15%)	57.6*	<0.001
**Female**	98 (47.57%)	102 (49.51%)	6 (2.91%)		
**Total**	121 (33.43%)	202 (55.8%)	39 (10.77%)		

* Statistically significant at 99% confidence.

HEPA, Health Enhancing Physical Activity.

### 3.3. Screen use

**Fig 1:**
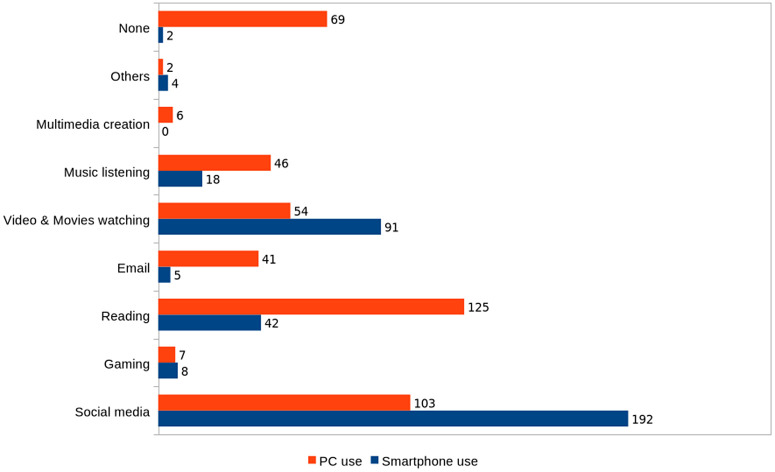
Common uses of gadgets in the participants (multiple response).

Out of the 357 participants that answered, all used computer gadgets of some form. The most commonly used gadgets were smartphone (329, 92.16%) and personal computer (PC, desktop or laptop) (170, 48.18%). More than half students (187, 51.66%) possessed multiple devices. Only 2 did not use smartphone while 69 did not use any PC regularly. The most common primary use of smartphone was scrolling some social media platform (192,53.78%), followed by watching videos and movies (91, 25.49%). PC was used most commonly for reading (125, 35%) and social media (103, 28.85%) (Fig 1).

**Fig 2:**
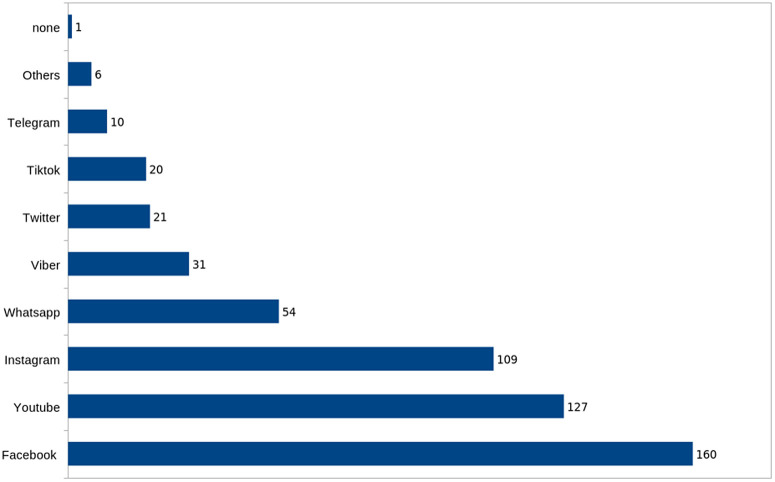
Frequency of common social media platforms used by participants (multiple response).

The most popular social media used by the students were Facebook/messenger (160, 44.82%) and YouTube (35.57%). Only one student reported not to use any social platform (Fig 2).

### 3.4. Daily device (screen) and internet use

The mean duration of any device (screen) use in the 357 students was 4.16 ± 1.88 (maximum 12) hours per day on weekdays, which rose to 6.36 ± 2.62 (maximum 16) hours per day on weekends and holidays. On an average, students were using internet over past 6.87 ± 3.33 years. Their daily internet consumption rate was 3.79 ± 2.03 (maximum 12) hours, which bumped to 5.79 ± 2.75 (maximum 18) hours on weekends and holidays. Eighty percent (289) students regularly looked at screen for more than 2 hours daily. Females used internet significantly longer than males every day (p = 0.01 for weekdays and 0.025 for weekend, Table 1).

### 3.5. Knowledge and utility of the devices

About a third (32.32%) of students had poor (nil to beginner) level of knowledge in computer system, but 56.25% expressed their high interest in learning it. Only a quarter of students were aware of the value of their private data in internet and practised securing their personal data regularly. About 85% participants found the internet “very useful” or “were dependent on it” for study (Table 4).

**Table 4:** Knowledge, interest and usefulness of computer technology.

Computer use parameters	Frequency	Percent
**Knowledge (n = 362)**		
Nil	17	4.70%
Beginner	100	27.62%
Working level	155	42.82%
Above average	73	20.17%
Very good	17	4.70%
**Interest (n = 352)**		
Not at all	3	0.85%
A little	44	12.50%
Moderate	107	30.40%
Much interested	164	46.59%
Eager to learn	34	9.66%
**Awareness of risk of privacy and practice of maintaining privacy in internet use (n = 352)**		
Not aware at all	22	6.25%
Heard, but not cared	13	3.69%
Heard, but not practised	87	24.72%
Aware and practise sometimes	142	40.34%
Well aware and practise regularly	88	25.00%
**Usefulness of internet in study (n = 352)**		
Nil	0	0.00%
A little	11	3.13%
Moderate	42	11.93%
Very much	243	69.03%
Dependent on internet	56	15.91%

Table 5 describes the correlation between multiple pairs of parameters of sleep, physical activity and internet use. It shows that amount of daily screen and internet use is positively correlated with higher sleep score (lower sleep quality). The amount of internet use in holidays is negatively correlated with total MET score over a week. Other pairs are not significantly correlated; notably, physical activity (MET value per week) is related neither to sleep score nor to daily screen use.

**Table 5:** Correlation of different health parameters.

Parameter1	Parameter2	Spearman Rho	*p* vale
Sleep Score	MET Value per week	-0.094	0.078
Sleep Score	Screen use on weekdays	0.217**	<0.001
Sleep Score	Screen use on holidays	0.131*	0.013
Sleep Score	Internet use on weekdays	0.143**	0.007
Sleep Score	Internet use on holidays	0.076	0.155
Total MET value	Screen use on weekdays	-0.062	0.246
Total MET value	Screen use on holidays	-0.049	0.357
Total MET value	Internet use on weekdays	-0.079	0.135
Total MET value	Internet use on holidays	-0.123*	0.02
PIU score	Annual family income	-0.044	0.476
PIU score	Sleep Score	0.270**	<0.001
PIU score	MET Value per week	-0.237**	<0.001
PIU score	Years of internet use	0.04	0.466
PIU score	Screen use on weekdays	0.252**	<0.001
PIU score	Screen use on holidays	0.281**	<0.001
PIU score	Internet use on weekdays	0.313**	<0.001
PIU score	internet use on holidays	0.302**	<0.001
PIU score‍	Knowledge in computer technology	-0.163**	0.010

Screen and internet use is in terms of hours per day.

* Significant correlation at 95% confidence level.

** Significant correlation at 99% confidence level.

### 3.6. Problematic internet use (PIU)

Among the three hundred fifty three students that responded to the PIU questionnaire completely, mean PIU score was 43.21 ± 9.28, with median 43 (IQR 37–50), ranging from 18 to 70. More than half (201, 56.94%) got the PIU score 42 or higher. The PIU score did not differ significantly between males and females (p = 0.565) (Table 1).

The PIU score was found to have significant positive correlation with sleep score and daily screen and internet use (p < 0.001). Although screen use is not correlated with physical activity, the PIU score correlated negatively with MET value per week of activity (Spearman rho -0.237, p < 0.001), meaning lower the physical activity in individuals, higher is the PIU score. It was not related to annual income and years of internet use (p > 0.05) (Table 5).

Multiple linear regression analysis was conducted to explore the predictors of problematic internet use (PIU) among the students. The model included age, sex (coded as 0 for female and 1 for male), income, sleep score, total weekly MET value and knowledge on computer, as predictors (Table 6). It demonstrated a statistically significant relationship with the PIU score as dependent variable (F(6, 261) = 7.14, p < 0.001). The overall model explained 14% of the variance in PIU scores (R^2^ = 0.14, adjusted R^2^ = 0.12).

**Table 6:** Regression analysis for predictors of PIU score.

Model Summary (PIU Score)						
R		R Square	Adjusted R Square		Std. Error of the Estimate	
0.38^a^		0.14	0.12		8.95	
^a^ Predictors: (Constant), Age, Sex, Income, Sleep Score, Total MET, Knowledge on computer						
**ANOVA (PIU score)**						
	Sum of Squares	df		Mean Square	F	P value
Regression	3426.96	6		571.16	7.14	<0.001*
Residual	20889.92	261		80.04		
Total	24316.88	267				
**Coefficients (PIU Score)**						
		Unstandard coefficients		Standard coefficients	T value	P value Beta
		Beta	Std error	Beta		
(constant)		38.54	5.44	.00	7.08	<0.001*
Age		.23	.23	.06	1.00	0.318
Sex**		3.20	1.22	.17	2.62	0.009*
Income		6.89E-007	3.12E-007	.13	2.21	0.028*
Sleep Score		.87	.23	.22	3.72	<0.001*
Total MET value		.00	.00	-.21	-3.33	0.001*
Knowledge on computer		-1.48	.60	-.15	-2.47	0.014*

* Significant at 95% CI.

** 1 for male and 0 for female.

Among the predictors, sex (β = 3.20, p = 0.009), sleep score (β = 0.87, p < 0.001), total MET (β = -0.21, p = 0.001), and knowledge of computer (β = -1.48, p = 0.014) emerged as significant predictors of PIU. Male students tend to have a higher PIU score by 3.2 units compared to females (significant at p = 0.009). Similarly, higher sleep score (signifying lower sleep quality) was associated with increased PIU scores. In contrast, higher levels of physical activity and higher tech knowledge were associated with lower PIU scores. Higher income levels were also associated with higher PIU scores (β = 6.89E-007, p = 0.028), indicating that family income may have minimal effect in the score. These findings suggest that sex, socioeconomic status, sleep habits, physical activity levels and knowledge on computer play roles in problematic internet use among undergraduate health-sciences students.

## 4. Discussion

This study highlights the patterns of digital device usage habits, sleep quality, physical activity levels, and problematic internet use (PIU) among undergraduate health-sciences students of Nepal. Focusing primarily on problematic internet use (PIU), we examined sleep and physical activity as key associated behaviours. Our analysis revealed several notable patterns and associations that have implications for understanding and addressing PIU in this population.

### 4.1. Sleep

Sleep patterns were examined in relation to problematic internet use (PIU), as excessive screen time may negatively impact sleep habits. Our study describes the sleep habits among undergraduate health-sciences students, revealing concerning trends in sleep duration and quality. While the median sleep duration was 7 hours, approximately one-third (30.45%) of participants reported inadequate sleep duration, consistent with findings in medical students of Kathmandu [22], which showed the figure to be 31.5%. However, our sample exhibited a lower proportion of overall poor sleep quality compared to other studies conducted in Nepal [11] and in India [23].

This discrepancy may be attributed to differences in the study population and the questionnaire tools. Our study was conducted across multiple centres, encompassing diverse geographic and demographic populations, whereas the aforementioned studies were limited to single-centre designs. Secondly, the composition of the study populations differed; our participants may have had varying socio-cultural, occupational, or environmental backgrounds, which can influence sleep behaviours and perceptions. Additionally, the use of different questionnaire tools across studies may have contributed to variations in reported sleep quality. Finally, geographical variation or seasonal variation may be responsible for the differences [24–26]. For instance, it can be assumed that people living in urban area have access to different recreational facilities that keep people indulging outside whereas it will not be possible in rural areas. Also, people tend to go to bed early and wake up late in winter season compared to summer. Our findings also show that women tend to have earlier sleep timing and longer sleep duration than men, consistent with previous studies, which have suggested that women have a shorter intrinsic circadian clock and perceive sleep quality differently from men [27–29].

Various studies have described the importance of sleep quality and quantity in different domains of life, including academic performance in students [12,30]. Oliver et al (2018) [31] demonstrated that sleep quality and duration was the best predictor for quality of life and perceived physical wellness in US college students. In our study, sleep quality was only one among the multiple health parameters related to computer use, and hence we did not use the full Pittsburgh sleep quality index. Future study could utilise it in a longitudinal study design, specially addressing sleep issues in wider population would yield a better picture about the gender difference in sleep parameters.

### 4.2. Physical activity

Physical activity was assessed as a key behaviour potentially associated with PIU and digital device use. Students having health-enhancing exercise were very low, and about one third were physically inactive, aligning with recent findings indicating insufficient physical activity among university students [32]. This also echoes global concerns, with WHO (2022) reporting that over 80% of adolescents and 27% of adults worldwide fail to meet recommended physical activity levels [33].

In contrast, a study based on nationwide Steps to Epidemiological Surveillance (STEPS) Survey data of 2013 in 4143 Nepalese adults of 15–69 years age using Global Physical Activity Questionnaire (GPAQ) has revealed that more than 96% have recommended levels of physical activity [34]. This high prevalence is not shared by other studies. For example, a GPAQ-based study in 945 high school students found that about 20% students had inadequate physical activity [35]. This discrepancy in prevalence of activity among the studies and our finding may be due to difference in sample population and the tools used. This might indicate a concerning trend of lower physical activity in young population, which might increase the proportion of non-communicable diseases in the future. Further investigation in different target groups of Nepalese population might provide better picture in this section.

Sex differences in physical activity were evident in our sample, with males having higher activity levels compared to females, consistent with previous research [36–38]. However, conflicting findings exist, as studies like Bertrand *et al* observed higher physical activity among females during the COVID-19 pandemic [39]. Luo and Zhong observed the differential trend of changing behaviours in US students about physical activity and sedentary life while emerging from adolescent to adulthood [40]. Thus there seems to be influence of multiple contextual factors, cultural norms, societal pressure and individual preferences that contribute to the apparent discrepancies in the gender difference in physical activity in our study.

The relationship between sleep quality or duration and physical activity has been a subject of interest in various studies. Studies have shown that physical activity has positive effect on sleep quality [41–43] and both physical activity and sound sleep have positive effect on health-related quality of life [44], and higher life satisfaction [45]. Our findings showed no significant correlation between these factors. Although the association between sleep and exercise have been discussed in multiple studies [46–51], the assumption that sleep quality or quantity can be enhanced solely through physical activity has been challenged [52–56]. This discrepancy is likely due to multiple confounders between these variables. Furthermore, studies typically take account of duration and time of sleep, not addressing its quality. It appears that physical activity and sedentary time have independent effect in sleep quality and/or quantity. Future study might utilize a separate investigation of sedentary time along with physical activity to comprehensively understand its effect in sleep quality as well as quantity.

### 4.3. Screen use

Students’ device screen use was evaluated not only as a behavioural pattern but also in its contribution to PIU and related health outcomes. Our study provides insights into the patterns and implications of screen use among undergraduate health-sciences students. While our findings indicate lower screen time than the global average [4], it is higher than studies in Indian population [57,58] and in Jumla, a remote place of Nepal [20]. This highlights significant reliance on digital devices among college students, particularly for social interaction and information-seeking purposes.

The association between screen time and various health outcomes is well-documented in literature, as excessive screen use is linked to poor sleep, cardiovascular diseases, stress, and psychological problems [7,9,57–61]. While this association varies across different context [60,62], a meta-analysis showed strong and consistent association between use of devices and reduced sleep quantity and quality, as well as increased daytime sleepiness [63]. Consistent with this, our report has shown the daily screen and internet use of the participants to be negatively correlated with sleep quality.

### 4.4. PIU

Our findings underline the growing burden of problematic internet use (PIU), a digitally mediated behavioural concern, among health-science undergraduates. In the context of increasing screen time, this aligns with global digital health priorities focused on behavioural health and digital addiction. It highlights the prevalence and correlates of problematic internet use (PIU) where more than half of participants had PIU. Prevalence of PIU in literature may differ with the population of study, time-frame and methodology, and ranges from 1.6 to 43.3% [11,20,9,64]. As noted in introduction section, PIU encompasses the problems of social, behavioural, and emotional issues, such as salience, excessive use, neglect of work, anticipation, lack of control, and neglect of social life. PIU is a broader term than internet addiction, which is characterised by loss of control and feeling of withdrawal [8,19]. Although we did use a cut-off value in the score for classification of PIU, it is essential to understand that that it is of less significance than the absolute score, as the goal is to minimize the score value.

In line with literature findings, our study demonstrates PIU score to be associated with higher sleep score, higher daily device and internet use and lower physical activity [50,58]. Although the PIU score was not significantly different between males and females in initial analysis, adjusting other factors shows being male is independently associated with a higher PIU score than being female. While some literature shows PIU, internet addiction or dependency to be more common in males [58,9,65] including in Nepali population [10,11], others report females to have higher PIU [22]. Such gender-mediated differences in internet addiction tendencies are reported to be due to economic factors, internet availability, social norms and some addiction-related health factors [66]. Further study on gender difference in PIU and its mediators can shed more light in this result. Nevertheless, these regression findings should be interpreted as exploratory, given the cross-sectional design and sample size constraints.

Use of portable devices means that users are almost constantly connected and exposed to the risk of its excess use. The anxiety related to fear of missing out (FOMO) is the psychological manifestation of dependency on the devices. It is also causally linked to psychological issues such as suicidal tendency, depression, negativity and cognitive impairment [7]. A high screen time also increases stress [67,68], affects students’ academic performance [69,70], and health-related quality of life [71]. In a study on students of Cairo, the sleep quality and academic performance were correlated negatively with night-time screen use, and positively with physical exercise [72]. Another study in China demonstrated that the combination of high screen time and insufficient vigorous physical activity was associated with the high prevalence of psychological problems such as depression, anxiety and school life dissatisfaction [73].

We found that socioeconomic status was significantly correlated with PIU, suggesting that students from higher socioeconomic background may be at higher risk. Our study also suggests that knowledge of computer technology may be a protective factor. A significant proportion of our students reported inadequate computer knowledge, but are very interested in learning it, while lower knowledge in technology is significantly associated with higher PIU score. Inadequate technical skills can impede students’ effective use of devices for studying, while excessive device use may also have detrimental effects on their academic performance. On the other hand, digital devices themselves can be used to monitor and regulate its excessive use. A study done at COVID-19 pandemic period has shown a positive relation in the use of fitness trackers and health-related mobile apps with the physical activity [74]. Another interventional study demonstrated that settings such as disabling non-essential notifications, switching the display to grayscale and other adjustments led to a reduction in problematic smartphone use and depressive symptoms, and in improving sleep quality [75]. These findings underscore the value of mindful gadget use—the conscious, purposeful engagement and deliberate disconnection—as an important approach to reducing problematic use and promoting healthier behavioural patterns. Thus, considering a high dependence on the internet reported by most of the our participants for their studies, it is important to balance the adequate digital knowledge and mindful device usage. Research suggests that mindfulness-based strategies, along with digital literacy and confidence in navigating digital environments, can further enhance well-being and mitigate the adverse effects of excessive screen time [76]. Also, given that digital health literacy and access to supportive tools vary widely, these findings have implications for equity in managing digitally-driven behavioural health challenges.

### 4.5. Recommendations

Based on these findings, targeted strategies can be designed to mitigate PIU and promote healthier sleep and physical activity behaviours. Recognizing the low physical activity level, inadequate sleep quality, high screen use and high PIU score among the students, it is important to implement appropriate intervention to the young adults. The sleep quality needs to be improved by incorporating sleep hygiene education and fostering healthy sleep habits in the context of increasing use of digital devices. The institutes also need to consider increasing physical activity opportunities on campus, integrate active breaks into academic schedules and offer fitness programs. Moreover, the negative correlation between computer-related knowledge and PIU implies that enhanced knowledge in computer technology may function as a protective factor against PIU. These findings also encourage the policymakers in training workshops on responsible internet use. These strategies support broader digital health goals among university students.

### 4.6. Limitations and future directions

As a cross-sectional design, this study precludes establishing causal relationships between the variables. Reliance on self-report measures introduces the potential for recall bias and social desirability effects, which can affect the validity and precision of the findings. They also remain vulnerable to subjective interpretation and reporting inconsistencies. The inclusion of objective measures – such as actigraphy for sleep or digital tracking for internet use – would enhance data accuracy, but was not feasible within the logistical and resource constraints of this study. The sample size was slightly below the target for prevalence estimation, and the study was not powered for association analyses; thus, related findings are exploratory. Sampling method was snowball, which tends to attract subjects with interest in the subject of investigation. Also, due to limited information, the sample could not be classified by programs of study for further analysis. Finally, while efforts were made to control for confounding variables, there may be unmeasured factors influencing the relationships between variables (such as mental health, lifestyle habits and pre-existing health conditions).

Future research should examine patterns of internet use and the prevalence of problematic internet use (PIU) across diverse populations. Longitudinal studies could provide further insights into the effects of excessive internet use on sleep quality among undergraduate health-sciences students. The influence of socioeconomic factors on PIU warrants additional exploration, alongside strategies to mitigate its impact. Further studies should also investigate the mechanisms by which knowledge in digital technology may reduce the risk of PIU, providing insights for intervention development and preventive efforts. A mixed-methods approach could enrich understanding by adding contextual depth and qualitative insight into health and behavioural dynamics. Finally, exploring the role of intervention strategies—including mindful use and self-regulation of the digital environment—would strengthen the digital health discourse in low-resource academic settings.

## 5. Conclusion

A high degree of physical inactivity, computer gadget use and problematic internet use have been observed in the undergraduate health-sciences students of Nepal. As Nepal’s technological progress exposes its youth to a rapidly evolving digital environment, unregulated use of computer gadgets may lead to unhealthy behavioural patterns. Interventions to promote sleep hygiene and physical activity, minimising screen use while maintaining adequate digital literacy and responsible internet use need to be implemented at personal and institutional levels. In this context, strategies encouraging self-regulation, such as awareness campaigns, behavioural counselling, mindful practices involving use of screen-time management tools, could support healthier digital engagement. Given the variation in digital health literacy and access to supportive interventions, our findings also highlight equity concerns in managing technology-mediated behavioural health risks. Findings should be interpreted with caution given the cross-sectional design and reliance on self-reported measures. Future prospective studies with more diverse samples could provide further insights into the complex relationship between PIU and its predictors.

## Supporting information

S1 File Questionnaire and pro-forma used to collect data in this study. (PDF)

S1 Data Master data (details of the data of individual participants of the study). (ODS)
