# Supplementary material for: Physical activity, sleep pattern and problematic internet use in undergraduate health-sciences students of Nepal – A cross-sectional study
Source: PLOS Digit Health. 2026 Jul 23;5(7):e0000797. doi: 10.1371/journal.pdig.0000797 (PMC13395320; doi:10.1371/journal.pdig.0000797)
Supplement: S1 File — (PDF) [file pdig.0000797.s001.pdf]

PHYSICAL ACTIVITY, SLEEP PATTERN AND SCREEN USAGE IN HEALTH-SCIENCE STUDENTS OF NEPAL – A CROSS-SECTIONAL STUDY

Please answer the following questions on your own. There is no right or wrong answer. We want to take your habit and opinion for health improvement. Your given data will not be shown to other people, without your permission.

Principle investigator: Dr Jay P Jha, KAHS (jay@kahs.edu.np)

Instruction to the participants:

1. Give your numerical answers as a SINGLE value. DO NOT give range.
2. Provide your answers based on current habits, usually within past 15 days.
3. Although each question is optional, we encourage you to attempt all the questions. If there is any negative answer, please mention it or write zero as your answer.

1. General Information

(Note: Personally identifiable information will be anonymised later)

School/College:
Age/Sex:
Email:
Address (City, District):

Email:
Phone:
Course:
Date:

Annual income of family (average): …………………………… Rs/year

Personal History:

Any long term disease you may have currently or had in past (please write the diagnosis, treatment, and current status. Eg, epilepsy for 2 years, under valproate, improving)………………………………………

Alcohol consumption: Never / Occasionally / Regularly / Dependent
Smoking: No / Yes

2. Sleep habit (Please answer about your current sleep pattern)

Average bed time:…………………… pm/am; wake-up time:……………………… am;
Duration of sleep:……………………hours [Please do not count any night duty schedules if you have]
It usually takes me ……………………. minutes to fall asleep.
I usually wake up ………………………. times during night.

Sleep disturbances (tick the boxes as applicable to you):

	Never or rarely	Occasionally	Frequently
I have trouble falling asleep			
Thoughts start racing while trying to sleep			
Frequent waking at night			
Difficulty returning to sleep after waking at night			
Early morning waking			
Snoring			
Excessive daytime sleepiness			

3. Physical activities:

Please include your average activity in past 7 days or more.

- ☐ Vigorous activities (like heavy exercise, heavy weight lifting, digging, aerobics, heavy outdoor sports like football, fast bicycling): ……………. minutes a day for ………………… days per week.
- ☐ Moderate physical activities (like jogging, light weight lifting, regular bicycling, light sports like table tennis, casual dance): ……………. minutes a day for ………………… days per week.
- ☐ Walking for at least 10 minutes at a time: ……………. minutes a day for ………… days per week.

4. Screen habits –

[Select your choices according to your current practice nowadays. Screen includes computer devices such as PC, laptop, mobile, tablet]

(a) Do you use any of the following devices? (circle all that you use regularly):
None / smartphone / laptop or desktop PC / tablet / Gaming consoles (PSP, Xbox) / Smart TV / Others (mention) ………………

(b) Whose device do you use regularly? My own / my family's / my friends'

(c) Do you have internet access? Yes / No

- If yes, how long have you been using internet? ……………………….. years

(d) *Average* duration of screen use …………………… hours per day on weekdays (Sunday to Friday), and ………………… hours on Saturday and holidays.

(e) *Average* duration of active internet use …………………….. hours/day on weekdays, and …………………hours on Saturday. *[Do not include time of online stay without actively using it]*

(f) *Most common* use of mobile (choose only one):
social media / gaming / reading / email / videos+movies / music / others………………….

(g) *Most common* use of computer [Laptop or PC] (choose only one):
social media / gaming / reading / email / videos+movies / No computer / others……………………………….

(h) *Primary* social media platform (Circle the SINGLE most frequent currently using platform only):
Twitter / facebook & messenger / Viber / Whatsapp / Instagram / Telegram / Tiktok / YouTube / None / Others ……………………..

(i) How much do you feel you have knowledge in computer technology?
Nil / Beginner / Working level / Above average / Very good

(j) How much is the internet useful in your studies?
None / a little / moderate / very much / I am dependent on it.

(k) How open are you to learning new things in computer technology?
No interest / a little interest / moderate interest / much interest / very eager to learn

(l) If the internet services, apps or websites ask for your personal information (like name, age, phone number, email), how much information do you like to give?

1. I give them whatever they ask happily. In return I get free services.
2. I give them partly and only when necessary
3. I give them fake information about me
4. I give only to some services which I have to use, but not to all.

5. I rarely give my personal info. I tend to avoid such apps or websites.

(m) How much are you bothered that your personal data might get leaked?

1. I do not care. I have nothing to hide.
2. I care overall only a little.
3. I care only if it gets leaked to wrong people who might misuse it.
4. I care if it gets leaked to public.
5. I am very careful about any data I enter in internet.

(n) How much are you aware about privacy and security in computer use?

1. Not aware at all
2. Heard about it but I do not care
3. Heard about it but do not know how to practice it
4. I am aware a little and practise it sometimes
5. I am well-aware and practise it regularly

5. Problematic Internet Use Questionnaire

Please answer following questions on your own with as much accuracy as possible. There is no right or wrong answer. For each question, tick in only one answer box out of five.

SN	Questions	Nev er	Rar ely	Somet imes	Ofte n	Alway s
1	How often does your internet use impair your work or study?					
2	How often do you want to decrease internet usage, but you cannot succeed?					
3	How often do you say “Just a couple minute more”?					
4	How often do you use internet in bed at sleep time?					
5	How often do you seek mobile phone when you wake up?					
6	How often do you feel you are missing some important notification in your devices?					
7	How often do you feel tense, irritated, or stressed if you cannot use internet for several days?					
8	How often do you feel tense, irritated, or stressed if you cannot use internet for as long as you want?					
9	How often do people, parents or friends complain to you about					

	spending too much time in internet?					
10	How often do you think of your device while working or studying?					
11	How often do you try to hide with others the duration you spend in internet?					
12	How often do you feel that your habit is causing problems to you?					
13	How often do you use internet instead of going out [with friends, to travel etc.]?					
14	How often do you neglect household works for using internet?					

Thank you for your time. We appreciate your involvement in this study. For more information about health and computer use, you may reach researcher of this study, Dr Jay at jay@kahs.edu.np.

In the end, please give us feedback about this survey. How do you feel about this questionnaire?

a) It is too long and boring
b) It is complex or confusing.
c) I am not interested in these things at all.
d) It is appropriate and of good length. I enjoyed filling the form.
e) It is too short. I can do more.

Any comments or feedback for making the study better:
