# Supplementary material for: Physical activity, sleep pattern and problematic internet use in undergraduate health-sciences students of Nepal – A cross-sectional study
Source: PLOS Digit Health. 2026 Jul 23;5(7):e0000797. doi: 10.1371/journal.pdig.0000797 (PMC13395320; doi:10.1371/journal.pdig.0000797)
Supplement: Attachment — Submitted filename: REVIEWER ATTACHMENT.docx [file pdig.0000797.s003.docx]

REVIEWER ATTACHMENT

Line 17 – Citation needed

Line 24 – How was the online questionnaire designed?

Line 33 – First define MET before using abbreviation

Line 80 – Define Internet dependence and operationalize it for the study

Line 102 – 106 – The sample selection needed to be explained in detail. How many provinces does Nepal have? How many medical schools per province? How did they arrive at six medical colleges? What proportion of MBBS, BDS, Nursing and paramedics were studied and why? What were the selection criteria? What were the exclusion criteria?

Line 119 – Define NSF

Line 131 – How were the students approached/ selected

Line 159 – Put the percentages of smokers, occasional drinkers, and heavy drinkers.

Line 180-182 – Put reference for the scoring pattern

Line 183 – Replace “in the 356 participants….” With “Among the 356 participants….”

Line 191-193 – Reconstruct the sentence to make a clearer sense.

Line 209 – Should read “Out of the 357 participants that answered”

Line 235 – Poo should be written correctly as poor

**NOTE:** No detailed Result section explanation given. It was only tables with minor explanation given. However, a lot of writings in the discussion should have been in the result section. Percentages should not appear in the discussion section, rather in the result section.

**Discussion** should only compare your findings with other research findings with an explanation on why they are supported or different from previous research findings.

Line 291 – Elaborate on difference in study population and questionnaire in relation to how they account for the discrepancy noted.

Line 292 – The basis for the assumption made here between urban and rural area does not appear logical as urban areas may have better income, better internet services and so on.

Line 316 – Abbreviation “STEPS” not defined or written in full first.

Line 365-369 – Explanation on PIU should go to the introduction part of the article.

Line 372-378 – Should be moved to the literature review part.
