# Supplementary material for: Physical activity, sleep pattern and problematic internet use in undergraduate health-sciences students of Nepal – A cross-sectional study
Source: PLOS Digit Health. 2026 Jul 23;5(7):e0000797. doi: 10.1371/journal.pdig.0000797 (PMC13395320; doi:10.1371/journal.pdig.0000797)
Supplement: Attachment — Submitted filename: REVIEWER_ATTACHMENT_auresp_1.docx [file pdig.0000797.s004.docx]

REVIEWER ATTACHMENT

Line 17 – Citation needed

Response: This is a part of abstract. We have not put any citation in abstract section. To encompass this content in Introduction section, we have modified a sentence in introduction, with citation.

Line 24 – How was the online questionnaire designed?

Response: We have described the details of questionnaire design in methodology section, and have not described in abstract. However, to address this comment, we have modified it in abstract as well.

Line 33 – First define MET before using abbreviation

Response: Meaning of MET added in the abstract. Thank you.

Line 80 – Define Internet dependence and operationalize it for the study

Response: The cited article (5) have used the study on internet dependence, which is not a parameter of our study. As it is close to PIU, which is our parameter, we have included the statement. Hence we feel it not necessary to “operationalize” for the study.

Line 102 – 106 – The sample selection needed to be explained in detail. How many provinces does Nepal have? How many medical schools per province? How did they arrive at six medical colleges? What proportion of MBBS, BDS, Nursing and paramedics were studied and why? What were the selection criteria? What were the exclusion criteria?

Response: Thank you for pointing this out. In Nepal, there are 7 provinces, out of which, one (province 7) had no medical college at the time of investigation. We chose one medical college from each of the remaining provinces as per convenience of approach to the students. All of them had most of the health science programs at undergraduate level – medical, dental, nursing, paramedical (medical laboratory tech, public health and pharmacy). The method of inclusion of the sample is by snowball approach. As our objective was not to compare the parameters in different programs of study, we did not include the proportionate data in the analysis. But we have updated the methods section to include this description. The inclusion and exclusion criteria are also mentioned.

Line 119 – Define NSF

Response: It is acronym for National Sleep Foundation. Included in the text, with citation.

Line 131 – How were the students approached/ selected

Response: We have explained this at lines 130-135 in Methods section. In short, we approached students via phone calls, email and social media. Questionnaire were distributed to them and they shared it among their peers. The initially approached student ensured the questionnaire form did not leak outside their college.

Line 159 – Put the percentages of smokers, occasional drinkers, and heavy drinkers.

Response: Done.

Line 180-182 – Put reference for the scoring pattern

Response: We have applied our own scoring system, based on criteria by National Sleep Foundation and other studies, as described in Methods section. The sleep score was directly compared in different groups, without applying cutoff points.

Line 183 – Replace “in the 356 participants….” With “Among the 356 participants….”

Response: Corrected.

Line 191-193 – Reconstruct the sentence to make a clearer sense.

Response: Corrected.

Line 209 – Should read “Out of the 357 participants that answered”

Response: Corrected.

Line 235 – Poo should be written correctly as poor

Response: Corrected.

**NOTE:** No detailed Result section explanation given. It was only tables with minor explanation given. However, a lot of writings in the discussion should have been in the result section. Percentages should not appear in the discussion section, rather in the result section.

**Discussion** should only compare your findings with other research findings with an explanation on why they are supported or different from previous research findings.

Response: We have structured the result section as per the author guideline of the journal. As data is presented in figures and tables, we did not include each values in its description, and only the essential findings are described in the result body.

We did not fully understand what portions of discussion should have been replaced in result section. In discussion, we have included some percent values only in such context as to compare with similar figures from other studies. We have not merely repeated the result findings in discussion; we included them only in context of comparing other studies and synthesizing its meaning. We think including those details in result section would not be appropriate. However, we have modified the discussion to omit percent values.

Line 291 – Elaborate on difference in study population and questionnaire in relation to how they account for the discrepancy noted.

Response: A single-centric studies like other studies cited has different population composition than ours (milticentric one). One of the differences can be seen in RCT, for example, where they have shown that single-center trials show larger treatment effects than multicenter ones (<https://pubmed.ncbi.nlm.nih.gov/21727292/>).

We also think the questionnaire tool used in the studies have some influence in the result, as each has different sensitivity of detecting the sleep quality. We did not include these statements, as we felt it was self-explanatory for a scientific person. However, we have updated the sentence and explained them to make it clearer.

Line 292 – The basis for the assumption made here between urban and rural area does not appear logical as urban areas may have better income, better internet services and so on.

Response: As stated, this is assumption, a personal opinion based on authors’ observation. Future research may address this hypothesis to confirm or refute it. The rationale given by reviewer may also seem valid, but the factors like better income and better internet services are not related to sleep quality. On the other hand, the factors such as indulging in recreation is logically related to sleep quality (and also to physical activity). So if the editor feels acceptable, we would like to keep the hypothesis here.

Line 316 – Abbreviation “STEPS” not defined or written in full first.

Response: The acronym "STEPS" stands for "Steps to Epidemiological Surveillance". It is a standardized method developed by the World Health Organization (WHO) to access the non-communicable disease (NCD) risk factors. The acronym is now explained in the text.

Line 365-369 – Explanation on PIU should go to the introduction part of the article.

Response: Thank you. We have updated the the text in Introduction and discussion part accordingly.

Line 372-378 – Should be moved to the literature review part.

Response: Thank you. We revised the text in Introduction accordingly.
