# Supplementary material for: Physical activity, sleep pattern and problematic internet use in undergraduate health-sciences students of Nepal – A cross-sectional study
Source: PLOS Digit Health. 2026 Jul 23;5(7):e0000797. doi: 10.1371/journal.pdig.0000797 (PMC13395320; doi:10.1371/journal.pdig.0000797)
Supplement: Attachment — Submitted filename: Response to Reviewers.doc [file pdig.0000797.s005.doc]

**REBUTTAL LETTER**

**Response to Decision Letter**

**Manuscript ID:** PDIG-D-25-00111R1

**Manuscript Title:** ­Physical activity, sleep pattern and problematic internet use in undergraduate health-science students of Nepal – a cross-sectional study

**Dear Reviewers and Editor of PLoS Digital Health,**

We sincerely thank you for the detailed, informative and thoughtful feedback, which has greatly helped us refine and strengthen this manuscript. Their constructive comments guided us to improve clarity, coherence, and scientific depth. We, all the authors have gone through each of the feedbacks and have incorporated it in the revised manuscript file. Following is the summary of the reviewers’ comments and our response in each.

| **S** | **Reviewers’ comments** | **Authors’ response** |
| --- | --- | --- |
| A | **REVIEWER 1** |  |
|  | The earlier observations made has been fully addressed by the authors and found to be satisfactory. The article meets the standard of PLOS Digital Health journal and should be accepted for publication. | Thank you. We appreciate that our manuscript has been found on par with the quality expected for PLoS Digital Health. |
| B | **REVIEWER 2** |  |
| 1. | Structure and Presentation: The manuscript could be strengthened in terms of narrative clarity and organisation. Although some progress has been made, the paper would benefit from a more coherent structure that guides the reader logically through the rationale, methods, results, and interpretation. As it stands, the flow is often disjointed, making it difficult to extract the central contributions of the work. I would strongly recommend a second round of restructuring, with particular attention to the sequencing and contextual framing of key points. Seeking feedback from colleagues outside their immediate field - but still within the broader academic environment - could be highly beneficial. Such readers can offer a fresh perspective on clarity, coherence, and narrative flow. This kind of cross-disciplinary input often highlights areas where communication could be improved for a broader scholarly audience. | Thank you for the comment. Our revised version of manuscript improves the narrative clarity and flow, ensuring a more coherent presentation of rationale, methods, results, and interpretation. |
| 2. | Use of English and Clarity of Expression: The authors have made progress in revising the manuscript, and as the content becomes more straightforward, the importance of polished and precise language stands out more prominently. At present, some grammatical inconsistencies and awkward phrasing occasionally make it difficult to follow the intended meaning, which can obscure the strength of the scientific contribution particularly for an international audience. A thorough language review - ideally by a fluent or native English speaker with experience in scientific writing - would improve the clarity and impact of the manuscript. | The revision has addressed the language mistakes, sentence structure and phrases. We also believe that scientific contributions should be assessed primarily on their content and rigour. We trust that the present revision conveys our findings clearly, and remain open to further editorial language support if deemed necessary. |
| 3. | Abstract and Framing: The abstract is quite difficult to follow. It lacks a structured format and does not clearly highlight the motivation, methods, findings, or implications of the work. I would suggest the authors consider following a standard scientific abstract structure, such as those found in Nature or outlined in guides like:  * https://mitcommlab.mit.edu/be/wp-content/uploads/sites/2/2016/08/journal-abstract1.gif  *https://books.openbookpublishers.com/10.11647/obp.0235/image/box_7_nature_abstract_writing.png  This would significantly improve the paper's accessibility and professional presentation. | The abstract has also been rewritten. We believe it now follows the “Nature” guidelines. |
| 4. | Incomplete References: The reference list at the end of the manuscript is currently cut off. Additionally, citations in the text do not appear to be properly linked. This should be corrected as a basic requirement for any scientific submission. | The issue of reference list seems to be because of automation by reference manager, and we apologise for this technical glitch. We have reviewed each reference manually and have ensured it follows the same format as the rest of manuscript. |
| 5. | Study Design and Limitations: Some of the limitations inherent in the study design should be clearly acknowledged, ideally both in the abstract and the introduction. Doing so provides important context for interpreting the results and aligns with expectations for transparency in biomedical publishing. | We have revised the limitation section in the main text, and also added in abstract and introduction. |
| 6. | External Feedback and Readability: Given the current challenges with flow and clarity, I would encourage the authors to seek informal feedback from a colleague unfamiliar with the paper. Such a reader could offer valuable insights into how the argument is unfolding (or failing to) and help improve the accessibility and logical structure of the manuscript. This type of narrative-focused feedback is different from the technical or statistical commentary that journal reviewers typically provide but is equally essential. | We acknowledge the reviewer’s suggestion and confirm that we have sought feedback from colleagues outside the immediate field. Their input has been incorporated into the present revision to improve consistency, readability and logical flow. We believe the manuscript is now clearer and more accessible for a broader audience. |
|  | Final remark: While the subject matter of the paper is potentially important and relevant to the scope of PLOS Digital Health, the current version still falls short of the standards expected for a top-tier biomedical journal. With another round of substantive revision - focused on restructuring, clarity, language and revisting all statistical methods - this paper could reach the level required for publication. Thank you for your hard work. | We appreciate the reviewer’s detailed engagement with our work. While some of the concerns raised were broad in scope, we have addressed them to the best extent possible within the aims and design of our study. We have substantially revised the manuscript with particular attention to structure, clarity, language, and statistical presentation, as suggested. We hope the revisions clarify the manuscript’s contribution and enhance its readability. We trust these revisions have strengthened the manuscript to meet the journal’s standards. |
| C | **REVIEWER 4** |  |
| 1. | **Comment No. 1**  Line 17-18: It suggests the results' argument, similar to line 41-43. It is advisable to remove it from the opening sentence of the abstract to bring clarity to results and article objectives. | Thank you. The abstract section has been rewritten. We like to point out that the line number in the comment does not match with that in the manuscript. This may be because of the formatting or other mismatch; we have tried to understand the comments by the reviewer as much as possible and have updated our manuscript according to suggestions. |
| 2. | **Comment No. 2**  Line 18-20: Sentence structure needs a clarity of purpose. Words redundancy should be avoided. In the beginning of sentence, it is 'Excessive Internet Usage' and in the later part, it is 'Excessive Internet Use' been used. | We reviewed the manuscript and found the inconsistency in use of the terms “use” and “usage”. As per my understanding, usage implies habitual use of certain tool or method, and the term “use” is more frequently used elsewhere. Hence we have changed the term “usage” to “use” throughout the manuscript except in some instances where the habit is implied (for example line 34 in abstract), and also in reference number 4. |
| 3. | **Comment No. 3**  The word "screen use" has been used that imply it in a general sense. However, later it is further defines as "excessive screen use'. There is a need to provide clarity on it. Either to use "screen use' only or the other throughout the manuscript. Both words implies different meaning/context to the manuscript. | We carefully checked each instance where “screen use” is mentioned throughout the manuscript. We are aware of the different implication when “excessive” term is used before it, and have mentioned it only when we intend to indicate the screen use is being excessive to affect their health or daily life; whereas ‘screen use’ is written in a general sense. We believe this distinction improves clarity and maintains consistency throughout the manuscript. |
| 4. | The sentence "by slightly adjusting the screen time and exercise " sounds vague. It needs to clarify what does it mean by adjustment (slightly) and exercise in the context (main manuscript). | Thank you. The vague phrase has been rephrased in the author summary. I hope the revision addresses this issue. |
| 5. | Consider replacing "PC" with full abbreviation "Personal Computer" | We replaced PC with personal computer and added PC as abbreviation in parentheses, so that the subsequent instances of PC gets understood. |
| 6. | Line 330: In the manuscript, the term "boys and 'girls' are used to refer to the population demographics. While the correlational study identifies the women having earlier sleep time owing to shorter circadian clock. The prospect of different population (age difference) must be added an acknowledgement. | As per reference 27 (Duffy et al, 2011), women had shorter circadian rhythm than men. They conducted the study in age 18 to 74 years, within which our sample also lies. Hence we feel no inconsistency to address here. To avoid confusion, the terms boys and girls are replaced with males and females [which resonates with the following comment as well]. |
| 7. | Consider replacing the term"girls" and "boys" with "young adults", "young women", "young men" to maintain consistency and appropriate age classification as per WHO age group classification. Boy/Girl term is common term used to identify age range of 0-19 years(infant, child & adolescent age) | Replaced boys with males and girls with females throughout the manuscript. |
| 8. | To ensure consistency in terminology, it is recommended to replace all instances of "University" with "college" throughout the manuscript. | Our study has been done in colleges, not universities, and the study findings are reported as college throughout the manuscript. The only two instances of “university” was used in reference to other studies which did use the university students in their study. Although we have no issue in naming it to college, we feel it would not be fair to change the other study’s terminology and have kept them as it is. |

Thank you.
