# Supplementary material for: Physical activity, sleep pattern and problematic internet use in undergraduate health-sciences students of Nepal – A cross-sectional study
Source: PLOS Digit Health. 2026 Jul 23;5(7):e0000797. doi: 10.1371/journal.pdig.0000797 (PMC13395320; doi:10.1371/journal.pdig.0000797)
Supplement: Attachment — Submitted filename: REVIEWER COMMENTS-Plos Digital Health-09.11.25.docx [file pdig.0000797.s006.docx]

**REVIEWER'S COMMENTS**

Thank you for the opportunity to review this paper titled ‘physical activity, sleep pattern and problematic internet use in undergraduate health-science students of Nepal – a cross-sectional study’.

**General comment**: It is a significant and relevant contribution of public health importance, as the authors explored the patterns of computer usage, sleep habits, and physical activity among

Undergraduate health science students in Nepal, and investigated the prevalence and predictors of problematic internet use (PIU) among them. The survey revealed a relationship that exist between screen usage, PIU, physical activity and sleep among Nepal students. It therefore recommend the need for healthier lifestyle choices in the era of digital education among undergraduate students to promote physical and emotional well-being as well as enhanced academic performance among college students.

**Specific comment:**

**Abstract**: This seems adequate but the authors need to address the following concerns;

1. Line 24-26. The authors should include the percentage in the following sentence. …*About a third (insert percent here ) reported inadequate sleep and a similar number were physically inactive (what percentage?), with female (insert percentage) less active than male (percentage).*
2. Line 27-28, change the word ‘sex’ to ‘gender’ in the statement or be specific of implicated gender (male or female)..*Multivariable analysis confirmed that sex, sleep quality*…..

**Introduction:** This was adequate, however, there are certain unreferenced sentences here. For example;

1. Line 67-68, *Nepal is also seeing a growing internet penetration rate of 51.6% in 2023, which is rising (currently 55.8% in mid-2025)*..no reference
2. Line 78-83, the entire definition and description of PIU has no reference. The authors should reference this appropriately.
3. Line 92, the authors used the word studies but cited only one study at the end of the sentence. See.. *Studies also underscore the correlation between periods*………..(12)

**Methods:** The study was a cross-sectional survey and authors tried to explain the details in the methods section. However, there is need to address the following observations;

1. The authors selected a medical college in one out of 7 provinces in Nepal using purposive sampling and target population were undergraduates in MBBS, BDS, Nursing and paramedical sciences programs. What are the courses that constituted paramedical sciences in the sampled population?
2. The authors said all the consenting undergraduate students of different streams were included in the study. What did the authors meant by different streams? How did the authors achieved this? What form of sampling method was used to ensure adequate and fair representation of different streams capturing students from different programs (MBBS, Nursing BDS and paramedical sciences)?
3. What is the total population of undergraduate students involving all listed courses at the time of the study? This was not stated. This is very necessary to determine if the number sampled could be a true representation of the students studying MBBS, BDS, Nursing and Paramedical sciences.
4. The authors appeared to use convenience sampling with snow ball technique in selecting respondents which has its own draw back in this type of survey. What is the rationale for the use of snow balling, when the target population are located in the same faculty in a single college?
5. The authors used social media or phone call to reach out to potential respondents. What forms of social media handle (Whatapp, Instagram, twitter (X) etc.) was used? This needs to be specified.
6. What did authors meant by approachable students? Who are these approachable students? Are they students’ representative from each programs? How were they identify by the researchers? Please clarify
7. How did authors got the phone contacts of respondents? Please clarify
8. Instrument: The authors used semi structured questionnaire which comprised of many abridged tools to fit into the context in this study. However, it is very essential that description of these tools be detailed and organized in such a way that each adapted instrument is exhaustively described including scoring adapted for the purpose of this study before the next tool for clarity. Also, the authors shifted some discussion on the tools again under result section which may not be necessary as they ought to focus mainly on the findings or results only here. The authors captured almost all the information concerning the tools but were not described in a single and well-coordinated manner under instruments for quick understanding by the readers. For example the authors adapted questions from the Pittsburgh sleep quality index to assess sleep habits and sleep disturbances and combined the score for the two (0-17) without first describing what questions were asked under sleep habit and sleep disturbances with examples of such questions and corresponding scoring used, so as to have a clear understanding of combined scoring range of (0-17) quoted. It means the questions on sleep habits are 3 items with a scoring score 0-1, while the sleep disturbances was a 7-item tool with its own scoring (0-2). There is need to explain this clearly under a single subheading sleep assessment. I suggest the authors used this approach under instrument:
a. Demographic: describe briefly the questions that were asked under this
b. Sleep assessment: put all the tools description including examples of questions that were asked to assess sleep habits and sleep disturbances along with scoring to have a clear understanding even without going to look for your questionnaire or tables of results.
c. Physical activity: described the tool used for physical activity here and scoring method adopted under here (1PAQ).
d. Digital device: this description is adequate.
e. Problematic Internet Use (PIU) tool: described everything about this tool here, give few examples of the questions and expected responses and scoring used. Reference studies that have used the abridged version being considered in this study, if any and state if any previous validation of this tool in this population in the past in Nepal. Though authors was cleared that adapted version was review by expert and refined accordingly.

**Ethics:** Was there any permission sought from the college/faculty/department following the ethics approval from the Ethical Review Board of Nepal Health Research Council?

**Results:** This section was presented logically with a clear understanding as represented in the tables of results. However, here are few observations;

1. the information on the statistics of respondents who completed the survey from each programs (MBBS, BDS, Nursing etc) was not included as part of the demographic description of study population, which could have provided further insights on the demographic and may be subjected to further sub-analysis using outcome variables in this study. This should be acknowledged as part of the study limitation.
2. Line 191, you may rewrite the sentence by adding percentage. See… *out of 358 respondents, about a third (n=109, 30.45%) got sleep less than 7 hours, and 4 (1.12%) had more than 9 hours….*
3. Line 193*, Nineteen (5.31%) students had prolonged sleep latency of more than 30 minutes…*
4. *Table 2, under sleep disturbances section, add percentage to each of the frequency by putting it in bracket after the frequency. E.g Trouble falling asleep never or rarely 159 (?%), occasionally 168 (?%)*
5. The sentence on the line 197-199 *(the sleep disturbances were assessed by 7 item questionnaire ……..two students did not respond to sleep disturbance questions)* should be moved to instrument under sleep. Focus should be on results/findings only here not description of tool anymore
6. Line 199, t*he early morning waking was the most common (insert percentage for specificity and clarity) sleep disturbances*. The result section is about frequency and percentage as different from discussion. The authors should take note of this.
7. There are multiple repetition of statements by the authors. For example line 201-204 where the authors were describing scoring of tools should expunged and moved appropriately to the method section under instrument. Focus should be on result interpretation only
8. Line 205, *Although male sleep time was more delayed and had lower sleep duration (? time) than female*…. The authors should quote supporting figure and percentage for easy understanding. This should be the approach to result writing.
9. Under physical activity, line 211-221 that is describing the physical activity tool should be moved to the method under instrument. The authors should describe the finding here. There is need to be consistent. Similarly, scoring and categorization of physical activity using IPAQ (Line 225-226) should be moved to instrument. The relevant statement should start from *more than half of of the participants were minimally active (55.8%*)…… *male were significantly more active than females (please add relevant figure and possible p value here)*
10. Line 234. *The most common primary use of smartphone was scrolling some social media platform (?percent), followed by watching video and movies (?percent)……*The authors should add percentage for clarity.
11. Problematic internet use (line 273-277). The statement in line 273-276 is a repetition and is not necessary under results. This should be expunged. Focus on your finding than tool description. The statement should start from the mean of the tool. Also, line 281-284 should include correlation figure or p value gotten for clarity. Any important findings worth reporting in the result section should be complete with relevant figure for understanding. While we know that readers can check others in the table presented, but certain parameter must be complete in the reporting prose.

**Discussion:** This was detailed enough as relevant studies were cited for comparison. Here are few observations made that need clarifications;

1. Line 324. *Our study was conducted across multiple centres*,….. what did authors meant by multiple centres when a single college was the study site? Please clarify
2. Line 406-409 is a repetition statement and may not be necessary. The authors can paraphrase the sentence if there is need to refer to this.
3. Limitations and future directions: This was described adequately. However, the authors should include the drawback of sampling method used in selecting respondents. This has a tendencies to attract only respondents who are interested in the subject of investigation excluding several other potential respondents that could have affected the findings of this study. The other limitation was mentioned under results section (see my observation).

**Conclusion:** It captured key findings, and the authors highlighted clears recommendations for further studies.
