# Supplementary material for: Physical activity, sleep pattern and problematic internet use in undergraduate health-sciences students of Nepal – A cross-sectional study
Source: PLOS Digit Health. 2026 Jul 23;5(7):e0000797. doi: 10.1371/journal.pdig.0000797 (PMC13395320; doi:10.1371/journal.pdig.0000797)
Supplement: Attachment — Submitted filename: Response to Reviewers 3.doc [file pdig.0000797.s007.doc]

**REBUTTAL LETTER**

**Response to Decision Letter**

**Manuscript ID:** PDIG-D-25-00111R2

**Manuscript Title:** ­Physical activity, sleep pattern and problematic internet use in undergraduate health-science students of Nepal – a cross-sectional study

**Dear Reviewers and Editor of PLoS Digital Health,**

We sincerely thank you for the detailed, informative and thoughtful feedback, which has greatly helped us refine and strengthen this manuscript. Their constructive comments guided us to improve clarity, coherence, and scientific depth. We, all the authors have gone through each of the feedbacks and have incorporated it in the revised manuscript file. Following is the summary of the reviewers’ comments and our response in each.

| **S** | **Reviewers’ comments** | **Authors’ response** |
| --- | --- | --- |
| A | **Reviewer 5** |  |
| 1 | Line 24-26. The authors should include the percentage in the following sentence. …*About a third (insert percent here)...* | Percentage included as per suggestion. |
| 2 | Line 27-28, change the word ‘sex’ to ‘gender’ in the statement or be specific of implicated gender (male or female)..*Multivariable analysis confirmed that sex, sleep quality…..* | We have consistently chosen the term sex against gender throughout the manuscript, as we described the comparison between male and female as a biological variable, and not the social implication of it. We request the reviewer and the editor to consider keeping the term sex in this context. However, if suggested, we are open to change it to gender in the manuscript. |
| 3 | Line 67-68, *Nepal is also seeing a growing internet penetration rate of 51.6% in 2023, which is rising (currently 55.8% in mid-2025)..*no reference | The sentence following this included the references (3,4), which are applicable to the two statements.  But now we have separated the sources as per statements, with data updated as per 2026. |
| 4 | Line 78-83, the entire definition and description of PIU has no reference. The authors should reference this appropriately. | The reference (8) is applicable for PIU, which has been kept at the end of the description of PIU. |
| 5 | Line 92, the authors used the word studies but cited only one study at the end of the sentence. See.. Studies also underscore the correlation between periods………..(12) | The language is now corrected to reflect singular reference. |
| 6 | The authors selected a medical college in one out of 7 provinces in Nepal using purposive sampling and target population were undergraduates in MBBS, BDS, Nursing and paramedical sciences programs. What are the courses that constituted paramedical sciences in the sampled population? | Paramedical courses constituted medical lab technology, pharmacy and public health, which are included in the text. |
| 7 | The authors said all the consenting undergraduate students of different streams were included in the study. What did the authors meant by different streams? How did the authors achieved this? What form of sampling method was used to ensure adequate and fair representation of different streams capturing students from different programs? | Different stream means different programs run in the institute (such as MBBS, nursing etc). Updated the language to avoid confusion. |
| 8 | What is the total population of undergraduate students involving all listed courses at the time of the study? This was not stated. This is very necessary to determine if the number sampled could be a true representation of the students studying MBBS, BDS, Nursing and Paramedical sciences. | According to Medical Education Commission of Nepal (2022), (https://mec.gov.np/en/category/notice?page=46), total number of students allocated in medical, paramedical and nursing programs were 4697 (Information added in the text). As there were >30% of incomplete response regarding the program of study by our respondents, we could not reliably distribute the sample in different programs for further analysis. |
| 9 | The authors appeared to use convenience sampling with snow ball technique in selecting respondents which has its own draw back in this type of survey. What is the rationale for the use of snow balling, when the target population are located in the same faculty in a single college? | Medical college in each province was selected purposively. Students within each college were enrolled using snowball sampling as students are easier to approach and include by this method.  As explained in the beginning of method section, we selected six medical colleges, each with different faculties. The target population were not located in same college. The rationale of using snowball sampling was to include the participants as much as possible in the institute, where the authors were not constantly present during the data collection. The online platforms were utilised for the purpose. |
| 10 | The authors used social media or phone call to reach out to potential respondents. What forms of social media handle (Whatapp, Instagram, twitter (X) etc.) was used? This needs to be specified. | We allowed the students to use their convenient platforms, which were not traced during data collection. Whatever the method of approach was, the respondents filled the same online form (jotform) and submitted in same platform.  As described in the result, most of them used facebook group of platform. |
| 11 | What did authors meant by approachable students? Who are these approachable students? Are they students’ representative from each programs? How were they identify by the researchers? Please clarify | Researchers communicated with students as per their personal communication. They were not the representative from each program, but they communicated with other students as per their personal communication. |
| 12 | How did authors got the phone contacts of respondents? Please clarify | Phone contact were not mandatory, but some students were contacted as per the personal communication. Some students were already within the phone circle of investigators since few months to years, which were utilised for this study. |
| 13 | Instrument: The authors used semi structured questionnaire…. | Thank you for the detailed instruction. We have adapted the instrument section as per the suggestion. Hope the correction addresses the concerns. |
| 14 | Ethics: Was there any permission sought from the college……….. | We have asked for permission from the colleges, but was considered unnecessary from the majority of institutes, given the parent organisation (Nepal health Research Council) already gave permission for the research, which is applicable nationwide. |
| 15 | the information on the statistics of respondents who completed the survey from each programs (MBBS, BDS, Nursing etc) was not included as part of the demographic description... | See response to comment 8 above. |
| The remaining comments of result section can be divided into two types: | | |
| 16 | Move some description of result section to method section. | Thank you for the suggestion. The appropriate parts have been adjusted in method section. |
| 17 | Add percentage, numbers and p values in the parentheses in the text description. | We did not include all the numerical information in the text so as to make the description concise, informative and meaningful. We thought repeating the information in different parts of result section were unnecessary. Hence we used the phrases such as one third, rather than 30.45%, which is already mentioned in the tables or the pictures. We also did not include Spearman rho or p values in the text, but mentioned the significant relation and their strength in it, so that readers will get more nuanced information from the table.  Although we have now incorporated the numbers in parentheses in the result text as per the suggestion, we request the editorial team to reconsider this issue and decide whether to accept the changes or keep the previous format. |
| 18 | *Our study was conducted across multiple centres,…..* what did authors meant by multiple centres when a single college was the study site? Please clarify. | This study is indeed a multicentric study covering the entire nation. Six medical colleges were selected, one from each province of Nepal, as described in the beginning of the methods section. |
| 19 | Line 406-409 is a repetition statement and may not be necessary. The authors can paraphrase the sentence if there is need to refer to this. | In our revised manuscript, the lines 406-409 refer to the section discussing our finding of low physical activity compared to a STEPS national survey, and comparing the findings with others such as the one based on GPAQ. It is possible that the line numbers are not in sync between us. We have gone through the discussion section to check any repetition statements and have updated accordingly. |
| 20 | Limitations and future directions: This was described adequately. However, the authors should include the drawback of ……… | Limitation section expanded as per suggestion. |

Thank you.
