# Supplementary material for: Physical activity, sleep pattern and problematic internet use in undergraduate health-sciences students of Nepal – A cross-sectional study
Source: PLOS Digit Health. 2026 Jul 23;5(7):e0000797. doi: 10.1371/journal.pdig.0000797 (PMC13395320; doi:10.1371/journal.pdig.0000797)
Supplement: Attachment — Submitted filename: Response to Reviewers 4.doc [file pdig.0000797.s008.doc]

**REBUTTAL LETTER**

**Response to Decision Letter**

**Manuscript ID:** PDIG-D-25-00111R3

**Manuscript Title:** ­Physical activity, sleep pattern and problematic internet use in undergraduate health-science students of Nepal – a cross-sectional study

**Revised title**: Physical activity, sleep pattern and problematic internet use in undergraduate health-sciences students of Nepal – a cross-sectional study

**Dear Reviewers and Editor of PLoS Digital Health,**

We thank you for the recent feedback. Although it is short, the suggestion on language and word selection has improved the manuscript in terms of clarity and accuracy. We have corrected them as follows:

| **S** | **Reviewers’ comments** | **Authors’ response** |
| --- | --- | --- |
| A | **Reviewer 4** |  |
| 1 | Lines 22, 103, 108, 114, 119, and throughout the manuscript: Consider replacing “undergraduate health-science students” with “undergraduate health-sciences students.” | The phrase has been changed to “undergraduate health-sciences students” including in the title. |
| 2 | Lines 88–90: Consider revising  “…showed that Nepal had the highest prevalence of PIU as per the Generalized Problematic Internet Use Scale-2 (GPIUS2) scale (12.6%) (9).”  to  “…showed that Nepal had the highest prevalence of PIU (12.6%) as per the Generalized Problematic Internet Use Scale-2 (GPIUS2) (9).” | The positioning of the percent has been shifted as per the suggestion. |

We hope this correction meets the requirement of the journal for publication. Please do not hesitate to contact me for further clarification or correction if needed.

**We also like to take this opportunity to correct the affiliation for three of us, who have changed our work place recently. We have changed them in the manuscript, and hope this will suffice for the formality.**

**I also like to remind you that the initial corresponding author has been changed from Hiramani Prasad Chaudhary to myself (Jay Prakash Jha) with my email for correspondence being** [**spring-jay.uncheck266@slmail.me**](mailto:spring-jay.uncheck266@slmail.me)

Thank you.

Sincerely,

Jay Prakash Jha

(Corresponding author)
