# Supplementary material for: Physical activity, sleep pattern and problematic internet use in undergraduate health-sciences students of Nepal – A cross-sectional study
Source: PLOS Digit Health. 2026 Jul 23;5(7):e0000797. doi: 10.1371/journal.pdig.0000797 (PMC13395320; doi:10.1371/journal.pdig.0000797)
Supplement: Peer review history

# Peer review: Decision Letter 0

**DOI:** 10.1371/journal.pdig.0000797.r001

**Authors:** Dukyong Yoon, Margaret Isioma Ojeahere

5 May 2025

PDIG-D-25-00111­Physical activity, sleep pattern and screen usage in undergraduate health-science students of Nepal – a cross-sectional studyPLOS Digital Health Dear Dr. Chaudhary, Thank you for submitting your manuscript to PLOS Digital Health. After careful consideration, we feel that it has merit but does not fully meet PLOS Digital Health's publication criteria as it currently stands. Therefore, we invite you to submit a revised version of the manuscript that addresses the points raised during the review process. Please submit your revised manuscript within 60 days Jul 04 2025 11:59PM. If you will need more time than this to complete your revisions, please reply to this message or contact the journal office at digitalhealth@plos.org. When you're ready to submit your revision, log on to https://www.editorialmanager.com/pdig/ and select the 'Submissions Needing Revision' folder to locate your manuscript file. Please include the following items when submitting your revised manuscript:* A rebuttal letter that responds to each point raised by the editor and reviewer(s). You should upload this letter as a separate file labeled 'Response to Reviewers'. This file does not need to include responses to any formatting updates and technical items listed in the 'Journal Requirements' section below.* A marked-up copy of your manuscript that highlights changes made to the original version. You should upload this as a separate file labeled 'Revised Manuscript with Track Changes'.* An unmarked version of your revised paper without tracked changes. You should upload this as a separate file labeled 'Manuscript'. If you would like to make changes to your financial disclosure, competing interests statement, or data availability statement, please make these updates within the submission form at the time of resubmission. Guidelines for resubmitting your figure files are available below the reviewer comments at the end of this letter. We look forward to receiving your revised manuscript. Kind regards, Margaret Isioma Ojeahere, M.D., MSc., FWACPAcademic EditorPLOS Digital Health Margaret OjeahereAcademic EditorPLOS Digital Health Leo Anthony CeliEditor-in-ChiefPLOS Digital Healthorcid.org/0000-0001-6712-6626 **Journal Requirements:** 1. In the online submission form, you indicated that Data will be available on request. All PLOS journals now require all data underlying the findings described in their manuscript to be freely available to other researchers, either 1. In a public repository, 2. Within the manuscript itself, or 3. Uploaded as supplementary information. This policy applies to all data except where public deposition would breach compliance with the protocol approved by your research ethics board. If your data cannot be made publicly available for ethical or legal reasons (e.g., public availability would compromise patient privacy), please explain your reasons by return email and your exemption request will be escalated to the editor for approval. Your exemption request will be handled independently and will not hold up the peer review process, but will need to be resolved should your manuscript be accepted for publication. One of the Editorial team will then be in touch if there are any issues. 2. Please upload a copy of Figure 1, 2 which you refer to in your text on page 13, 14. Or, if the figure is no longer to be included as part of the submission please remove all reference to it within the text. 3. Please provide separate figure files in .tif or .eps format. For more information about figure files please see our guidelines:  https://journals.plos.org/digitalhealth/s/figures https://journals.plos.org/digitalhealth/s/figures#loc-file-requirements**Additional Editor Comments (if provided):** There are concerns that the study may not fall within the scope of digital health. Nonetheless, it appears to fall within the intended scope of PLOS Digital Health- "This community-driven journal reflects the full breadth of the digital health research environment, including social and ethical issues, with a strong focus on public health and big data management; including but not limited to, e-health, medical imaging and diagnostics, fairness and health disparities as outcomes from digital health research, patient accessibility and behavior, and precision medicine".

The study in its present form explores key aspects of digital health, particularly by examining behavioral patterns shaped by technology. It focuses on computer gadget usage, sleep habits, and physical activity, aligning with PLOS Digital Health’s scope, which includes public health, data management, patient behavior, and some health disparities.

However, I recommend that the authors emphasize the study's contributions to digital health discourse, potential technological interventions, and broader public health implications. I suggest that they incorporate insights from their findings, highlighting how e-health interventions or digital tools can impact student health, behavioral trends, and disparities and provide recommendations that align with these considerations.[Note: HTML markup is below. Please do not edit.]**Reviewers' Comments:**  Reviewer's Responses to Questions

**Comments to the Author**

1. Does this manuscript meet PLOS Digital Health’s publication criteria? Is the manuscript technically sound, and do the data support the conclusions? The manuscript must describe methodologically and ethically rigorous research with conclusions that are appropriately drawn based on the data presented.

Reviewer #1: No

Reviewer #2: Yes

Reviewer #3: No

**********

2. Has the statistical analysis been performed appropriately and rigorously?

Reviewer #1: No

Reviewer #2: Yes

Reviewer #3: Yes

**********

3. Have the authors made all data underlying the findings in their manuscript fully available (please refer to the Data Availability Statement at the start of the manuscript PDF file)?

The PLOS Data policy requires authors to make all data underlying the findings described in their manuscript fully available without restriction, with rare exception. The data should be provided as part of the manuscript or its supporting information, or deposited to a public repository. For example, in addition to summary statistics, the data points behind means, medians and variance measures should be available. If there are restrictions on publicly sharing data—e.g. participant privacy or use of data from a third party—those must be specified.

Reviewer #1: No

Reviewer #2: No

Reviewer #3: No

**********

4. Is the manuscript presented in an intelligible fashion and written in standard English?

PLOS Digital Health does not copyedit accepted manuscripts, so the language in submitted articles must be clear, correct, and unambiguous. Any typographical or grammatical errors should be corrected at revision, so please note any specific errors here.

Reviewer #1: Yes

Reviewer #2: Yes

Reviewer #3: Yes

**********

5. Review Comments to the Author

Please use the space provided to explain your answers to the questions above. You may also include additional comments for the author, including concerns about dual publication, research ethics, or publication ethics. (Please upload your review as an attachment if it exceeds 20,000 characters)

Reviewer #1: The title does not seem to capture entire aim of the article. It missed out on Problematic Internet Use which is a major variable in the study. I suggest that it be restructured as "Patterns of computer Usage, sleep habits and Problematic Internet Usage among health science students in Nepal".

Reviewer #2: General comments that summarise what needs to be added or strengthened for the paper to be considered publication-worthy:

1. Clarity and definition of MET term

The abstract mentions ‘MET score’ without providing a definition for its use. As this term refers to ‘Metabolic Equivalent’, which may not be familiar to all readers, it is essential to define it clearly in the abstract itself. This would help readers understand the relevance of this measure to the study’s objectives and findings.

2. Analytical Statistics

The paper refers to ‘analytical statistics’ but does not elaborate on what specific methods were used under this category. It would be helpful to specify which statistical tests or models were applied (it should be regression analysis, ANOVA, chi-square tests from my understanding), and why these were appropriate for the type of data collected. Clearer articulation of the analytical approach would enhance the transparency and reproducibility of the research.

3. Methodological rigour and consideration of confounding variables

While the study attempts to correlate screen time with sleep patterns and physical health indicators, it does not adequately address potential confounding factors. Screen time and sleep are influenced by numerous variables, such as socioeconomic status, mental health, lifestyle habits, and pre-existing conditions, that appear to be overlooked in the analysis. The authors should discuss these limitations more explicitly and consider either adjusting for these variables or acknowledging them as potential sources of bias.

4. Questionnaire use and limitations

The use of self-reported questionnaires is practical, but inherently limited by recall bias and subjectivity. The manuscript would benefit from a more extended discussion on how these limitations may have impacted the findings. Additionally, incorporating a mixed-methods approach or validating the questionnaire with objective data (e.g., wearable devices) could strengthen future iterations of the study.

5. Citations and framing

The mention of the National Institute of Health requires further elaboration. More information about the institute's recommendations needed. Also, these recommendations are probably outdated.

6. Sample size justification

The manuscript mentions a minimum sample size but does not provide sufficient explanation on how this was calculated or justified except for the confidence level used. It is important to detail the statistical rationale (e.g., power analysis, effect size) behind determining the sample size to affirm the study's validity and generalisability.

7. Parameter explanation

All key parameters and variables used in the study should be clearly defined and explained in both the methods and results sections. This includes units of measurement and any thresholds used to interpret results (e.g., what constitutes "high" vs. "low" screen time or poor vs. good sleep quality).

Reviewer #3: Authors attempted to evaluate the use of the internet usage/screen time on physical activities and sleep using a set of questionnaires deployed via a web-based portal. Their approach to analyzing the data was thoughtful and thorough. Results are plausible. The discussion is adequate.

**********

6. PLOS authors have the option to publish the peer review history of their article (what does this mean?). If published, this will include your full peer review and any attached files.

**Do you want your identity to be public for this peer review?**  If you choose “no”, your identity will remain anonymous but your review may still be made public.

For information about this choice, including consent withdrawal, please see our Privacy Policy.

Reviewer #1: **Yes:** NWOGA CHARLES NNAEMEKA

Reviewer #2: No

Reviewer #3: No

**********

[NOTE: If reviewer comments were submitted as an attachment file, they will be attached to this email and accessible via the submission site. Please log into your account, locate the manuscript record, and check for the action link "View Attachments". If this link does not appear, there are no attachment files.]**Figure resubmission:** While revising your submission, please upload your figure files to the Preflight Analysis and Conversion Engine (PACE) digital diagnostic tool, https://pacev2.apexcovantage.com/. PACE helps ensure that figures meet PLOS requirements. To use PACE, you must first register as a user. Registration is free. Then, login and navigate to the UPLOAD tab, where you will find detailed instructions on how to use the tool. If you encounter any issues or have any questions when using PACE, please email PLOS at figures@plos.org. Please note that Supporting Information files do not need this step. If there are other versions of figure files still present in your submission file inventory at resubmission, please replace them with the PACE-processed versions. **Reproducibility:** To enhance the reproducibility of your results, we recommend that authors of applicable studies deposit laboratory protocols in protocols.io, where a protocol can be assigned its own identifier (DOI) such that it can be cited independently in the future. Additionally, PLOS ONE offers an option to publish peer-reviewed clinical study protocols. Read more information on sharing protocols at https://plos.org/protocols?utm_medium=editorial-email&utm_source=authorletters&utm_campaign=protocols

Attachment Submitted filename: REVIEWER ATTACHMENT.docx

# Author comment: Author response to Decision Letter 1

**DOI:** 10.1371/journal.pdig.0000797.r002

15 Jun 2025

Attachment Submitted filename: REVIEWER_ATTACHMENT_auresp_1.docx

# Peer review: Decision Letter 1

**DOI:** 10.1371/journal.pdig.0000797.r003

**Authors:** Dukyong Yoon, Margaret Isioma Ojeahere

15 Aug 2025

PDIG-D-25-00111R1­Physical activity, sleep pattern and problematic internet use in undergraduate health-science students of Nepal – a cross-sectional studyPLOS Digital Health Dear Dr. Jha, Thank you for submitting your manuscript to PLOS Digital Health. After careful consideration, we feel that it has merit but does not fully meet PLOS Digital Health's publication criteria as it currently stands. Therefore, we invite you to submit a revised version of the manuscript that addresses the points raised during the review process. Please submit your revised manuscript within 30 days Sep 14 2025 11:59PM. If you will need more time than this to complete your revisions, please reply to this message or contact the journal office at digitalhealth@plos.org. When you're ready to submit your revision, log on to https://www.editorialmanager.com/pdig/ and select the 'Submissions Needing Revision' folder to locate your manuscript file. Please include the following items when submitting your revised manuscript:* A rebuttal letter that responds to each point raised by the editor and reviewer(s). You should upload this letter as a separate file labeled 'Response to Reviewers'. This file does not need to include responses to any formatting updates and technical items listed in the 'Journal Requirements' section below.* A marked-up copy of your manuscript that highlights changes made to the original version. You should upload this as a separate file labeled 'Revised Manuscript with Track Changes'.* An unmarked version of your revised paper without tracked changes. You should upload this as a separate file labeled 'Manuscript'. If you would like to make changes to your financial disclosure, competing interests statement, or data availability statement, please make these updates within the submission form at the time of resubmission. Guidelines for resubmitting your figure files are available below the reviewer comments at the end of this letter. We look forward to receiving your revised manuscript. Kind regards, Margaret Isioma Ojeahere, M.D., MSc., FWACPAcademic EditorPLOS Digital Health Margaret OjeahereAcademic EditorPLOS Digital Health Leo Anthony CeliEditor-in-ChiefPLOS Digital Healthorcid.org/0000-0001-6712-6626  **Journal Requirements:** 1. If the reviewer comments include a recommendation to cite specific previously published works, please review and evaluate these publications to determine whether they are relevant and should be cited. There is no requirement to cite these works unless the editor has indicated otherwise.  **Additional Editor Comments (if provided):**    [Note: HTML markup is below. Please do not edit.] **Reviewers' Comments:**  Reviewer's Responses to Questions

**Comments to the Author**

1. If the authors have adequately addressed your comments raised in a previous round of review and you feel that this manuscript is now acceptable for publication, you may indicate that here to bypass the “Comments to the Author” section, enter your conflict of interest statement in the “Confidential to Editor” section, and submit your "Accept" recommendation.

Reviewer #1: All comments have been addressed

Reviewer #2: (No Response)

Reviewer #4: (No Response)

**********

2. Does this manuscript meet PLOS Digital Health’s publication criteria? Is the manuscript technically sound, and do the data support the conclusions? The manuscript must describe methodologically and ethically rigorous research with conclusions that are appropriately drawn based on the data presented.

Reviewer #1: Yes

Reviewer #2: Partly

Reviewer #4: Yes

**********

3. Has the statistical analysis been performed appropriately and rigorously?

Reviewer #1: Yes

Reviewer #2: I don't know

Reviewer #4: Yes

**********

4. Have the authors made all data underlying the findings in their manuscript fully available (please refer to the Data Availability Statement at the start of the manuscript PDF file)?

The PLOS Data policy requires authors to make all data underlying the findings described in their manuscript fully available without restriction, with rare exception. The data should be provided as part of the manuscript or its supporting information, or deposited to a public repository. For example, in addition to summary statistics, the data points behind means, medians and variance measures should be available. If there are restrictions on publicly sharing data—e.g. participant privacy or use of data from a third party—those must be specified.

Reviewer #1: Yes

Reviewer #2: Yes

Reviewer #4: No

**********

5. Is the manuscript presented in an intelligible fashion and written in standard English?

PLOS Digital Health does not copyedit accepted manuscripts, so the language in submitted articles must be clear, correct, and unambiguous. Any typographical or grammatical errors should be corrected at revision, so please note any specific errors here.

Reviewer #1: Yes

Reviewer #2: Yes

Reviewer #4: Yes

**********

6. Review Comments to the Author

Please use the space provided to explain your answers to the questions above. You may also include additional comments for the author, including concerns about dual publication, research ethics, or publication ethics. (Please upload your review as an attachment if it exceeds 20,000 characters)

Reviewer #1: The earlier observations made has been fully addressed by the authors and found to be satisfactory. The article meets the standard of PLOS Digital Health journal and should be accepted for publication.

Reviewer #2: I appreciate the authors' efforts in revising the manuscript and addressing prior feedback. While there has been some improvements, I still have concerns regarding the overall structure and clarity of the paper, which make it difficult to fully appreciate the underlying content and results. Below is a more detailed discussion for your reference.

1. Structure and Presentation: The manuscript could be strengthened in terms of narrative clarity and organisation. Although some progress has been made, the paper would benefit from a more coherent structure that guides the reader logically through the rationale, methods, results, and interpretation. As it stands, the flow is often disjointed, making it difficult to extract the central contributions of the work. I would strongly recommend a second round of restructuring, with particular attention to the sequencing and contextual framing of key points. Seeking feedback from colleagues outside their immediate field - but still within the broader academic environment - could be highly beneficial. Such readers can offer a fresh perspective on clarity, coherence, and narrative flow. This kind of cross-disciplinary input often highlights areas where communication could be improved for a broader scholarly audience.

2. Use of English and Clarity of Expression: The authors have made progress in revising the manuscript, and as the content becomes more straightforward, the importance of polished and precise language stands out more prominently. At present, some grammatical inconsistencies and awkward phrasing occasionally make it difficult to follow the intended meaning, which can obscure the strength of the scientific contribution particularly for an international audience. A thorough language review - ideally by a fluent or native English speaker with experience in scientific writing - would improve the clarity and impact of the manuscript.

3. Abstract and Framing: The abstract is quite difficult to follow. It lacks a structured format and does not clearly highlight the motivation, methods, findings, or implications of the work. I would suggest the authors consider following a standard scientific abstract structure, such as those found in Nature or outlined in guides like:

* https://mitcommlab.mit.edu/be/wp-content/uploads/sites/2/2016/08/journal-abstract1.gif

* https://books.openbookpublishers.com/10.11647/obp.0235/image/box_7_nature_abstract_writing.png

This would significantly improve the paper's accessibility and professional presentation.

4. Incomplete References: The reference list at the end of the manuscript is currently cut off. Additionally, citations in the text do not appear to be properly linked. This should be corrected as a basic requirement for any scientific submission.

5. Study Design and Limitations: Some of the limitations inherent in the study design should be clearly acknowledged, ideally both in the abstract and the introduction. Doing so provides important context for interpreting the results and aligns with expectations for transparency in biomedical publishing.

6. External Feedback and Readability: Given the current challenges with flow and clarity, I would encourage the authors to seek informal feedback from a colleague unfamiliar with the paper. Such a reader could offer valuable insights into how the argument is unfolding (or failing to) and help improve the accessibility and logical structure of the manuscript. This type of narrative-focused feedback is different from the technical or statistical commentary that journal reviewers typically provide but is equally essential.

Final remark: While the subject matter of the paper is potentially important and relevant to the scope of PLOS Digital Health, the current version still falls short of the standards expected for a top-tier biomedical journal. With another round of substantive revision - focused on restructuring, clarity, language and revisting all statistical methods - this paper could reach the level required for publication. Thank you for your hard work.

Reviewer #4: Line 17-18: It suggests the results' argument, similar to line 41-43. It is advisable to remove it from the opening sentence of the abstract to bring clarity to results and article objectives.

Line 18-20: Sentence structure needs a clarity of purpose. Words redundancy should be avoided. In the beginning of sentence, it is 'Excessive Internet Usage' and in the later part, it is 'Excessive Internet Use' been used.

Line 51: Replace "We have shown that..." with "It is evident in the study that..."

Line 54: Replace "The result highlights the..." with "The following study findings highlight the"

Line 56-57: The word "screen use" has been used that imply it in a general sense. However, later it is further defines as "excessive screen use'. There is a need to provide clarity on it. Either to use "screen use' only or the other throughout the manuscript. Both words implies different meaning/context to the manuscript.

Line 57-58: The sentence "by slightly adjusting the screen time and exercise " sounds vague. It needs to clarify what does it mean by adjustment (slightly) and exercise in the context (main manuscript).

Line 112: Replace "'This finding' with "This study finding"

Line 117: Replace "This was a cross-sectional study" with "Ä cross-sectional study"

Line 204: Replace "sheep' with "sleep"

Line 217-219: Replace "number day in week" with "number of days in week"

Line 232: Consider replacing "PC" with full abbreviation "Personal Computer"

Line 330: In the manuscript, the term "boys and 'girls' are used to refer to the population demographics. While the correlational study identifies the women having earlier sleep time owing to shorter circadian clock. The prospect of different population (age difference) must be added an acknowledgement.

Line 207, 225, 228, 253, 280, 330: Consider replacing the term"girls" and "boys" with "young adults", "young women", "young men" to maintain consistency and appropriate age classification as per WHO age group classification. Boy/Girl term is common term used to identify age range of 0-19 years(infant, child & adolescent age)

Line 346: To ensure consistency in terminology, it is recommended to replace all instances of "University" with "college" throughout the manuscript.

Line 454: Replace "This study" with "This study findings"

**********

7. PLOS authors have the option to publish the peer review history of their article (what does this mean?). If published, this will include your full peer review and any attached files.

**Do you want your identity to be public for this peer review?**  If you choose “no”, your identity will remain anonymous but your review may still be made public.

For information about this choice, including consent withdrawal, please see our Privacy Policy.

Reviewer #1: **Yes:** Nwoga Charles Nnaemeka

Reviewer #2: No

Reviewer #4: **Yes:** Seemab Mehmood

**********

[NOTE: If reviewer comments were submitted as an attachment file, they will be attached to this email and accessible via the submission site. Please log into your account, locate the manuscript record, and check for the action link "View Attachments". If this link does not appear, there are no attachment files.] **Figure resubmission:**  While revising your submission, please upload your figure files to the Preflight Analysis and Conversion Engine (PACE) digital diagnostic tool, https://pacev2.apexcovantage.com/. PACE helps ensure that figures meet PLOS requirements. To use PACE, you must first register as a user. Registration is free. Then, login and navigate to the UPLOAD tab, where you will find detailed instructions on how to use the tool. If you encounter any issues or have any questions when using PACE, please email PLOS at figures@plos.org. Please note that Supporting Information files do not need this step. If there are other versions of figure files still present in your submission file inventory at resubmission, please replace them with the PACE-processed versions. **Reproducibility:**  To enhance the reproducibility of your results, we recommend that authors of applicable studies deposit laboratory protocols in protocols.io, where a protocol can be assigned its own identifier (DOI) such that it can be cited independently in the future. Additionally, PLOS ONE offers an option to publish peer-reviewed clinical study protocols. Read more information on sharing protocols at https://plos.org/protocols?utm_medium=editorial-email&utm_source=authorletters&utm_campaign=protocols

# Author comment: Author response to Decision Letter 2

**DOI:** 10.1371/journal.pdig.0000797.r004

10 Sep 2025

Attachment Submitted filename: Response to Reviewers.doc

# Peer review: Decision Letter 2

**DOI:** 10.1371/journal.pdig.0000797.r005

**Authors:** Dukyong Yoon, Margaret Isioma Ojeahere

25 Nov 2025

PDIG-D-25-00111R2­Physical activity, sleep pattern and problematic internet use in undergraduate health-science students of Nepal – a cross-sectional studyPLOS Digital Health Dear Dr. Jha, Thank you for submitting your manuscript to PLOS Digital Health. After careful consideration, we feel that it has merit but does not fully meet PLOS Digital Health's publication criteria as it currently stands. Therefore, we invite you to submit a revised version of the manuscript that addresses the points raised during the review process. Please submit your revised manuscript by Jan 24 2026 11:59PM. If you will need more time than this to complete your revisions, please reply to this message or contact the journal office at digitalhealth@plos.org. When you're ready to submit your revision, log on to https://www.editorialmanager.com/pdig/ and select the 'Submissions Needing Revision' folder to locate your manuscript file. Please include the following items when submitting your revised manuscript:* A rebuttal letter that responds to each point raised by the editor and reviewer(s). You should upload this letter as a separate file labeled 'Response to Reviewers'. This file does not need to include responses to any formatting updates and technical items listed in the 'Journal Requirements' section below.* A marked-up copy of your manuscript that highlights changes made to the original version. You should upload this as a separate file labeled 'Revised Manuscript with Track Changes'.* An unmarked version of your revised paper without tracked changes. You should upload this as a separate file labeled 'Manuscript'. If you would like to make changes to your financial disclosure, competing interests statement, or data availability statement, please make these updates within the submission form at the time of resubmission. Guidelines for resubmitting your figure files are available below the reviewer comments at the end of this letter. We look forward to receiving your revised manuscript. Kind regards, Margaret Isioma Ojeahere, M.D., MSc., FWACPAcademic EditorPLOS Digital Health Margaret OjeahereAcademic EditorPLOS Digital Health Leo Anthony CeliEditor-in-ChiefPLOS Digital Healthorcid.org/0000-0001-6712-6626 **Journal Requirements:**  If the reviewer comments include a recommendation to cite specific previously published works, please review and evaluate these publications to determine whether they are relevant and should be cited. There is no requirement to cite these works unless the editor has indicated otherwise.  **Additional Editor Comments (if provided):**  I recommend that the authors enhance the quality of the submitted article by addressing the reviewers' suggestions, and improving its grammar, flow, and overall organization. [Note: HTML markup is below. Please do not edit.] **Reviewers' Comments:**  Reviewer's Responses to Questions

**Comments to the Author**

1. If the authors have adequately addressed your comments raised in a previous round of review and you feel that this manuscript is now acceptable for publication, you may indicate that here to bypass the “Comments to the Author” section, enter your conflict of interest statement in the “Confidential to Editor” section, and submit your "Accept" recommendation.

Reviewer #2: All comments have been addressed

Reviewer #4: All comments have been addressed

Reviewer #5: All comments have been addressed

**********

2. Does this manuscript meet PLOS Digital Health’s publication criteria? Is the manuscript technically sound, and do the data support the conclusions? The manuscript must describe methodologically and ethically rigorous research with conclusions that are appropriately drawn based on the data presented.

Reviewer #2: Partly

Reviewer #4: Yes

Reviewer #5: Yes

**********

3. Has the statistical analysis been performed appropriately and rigorously?

Reviewer #2: I don't know

Reviewer #4: Yes

Reviewer #5: Yes

**********

4. Have the authors made all data underlying the findings in their manuscript fully available (please refer to the Data Availability Statement at the start of the manuscript PDF file)?

The PLOS Data policy requires authors to make all data underlying the findings described in their manuscript fully available without restriction, with rare exception. The data should be provided as part of the manuscript or its supporting information, or deposited to a public repository. For example, in addition to summary statistics, the data points behind means, medians and variance measures should be available. If there are restrictions on publicly sharing data—e.g. participant privacy or use of data from a third party—those must be specified.

Reviewer #2: No

Reviewer #4: Yes

Reviewer #5: Yes

**********

5. Is the manuscript presented in an intelligible fashion and written in standard English?

PLOS Digital Health does not copyedit accepted manuscripts, so the language in submitted articles must be clear, correct, and unambiguous. Any typographical or grammatical errors should be corrected at revision, so please note any specific errors here.

Reviewer #2: Yes

Reviewer #4: Yes

Reviewer #5: Yes

**********

6. Review Comments to the Author

Please use the space provided to explain your answers to the questions above. You may also include additional comments for the author, including concerns about dual publication, research ethics, or publication ethics. (Please upload your review as an attachment if it exceeds 20,000 characters)

Reviewer #2: Thank you for your improvements to the manuscript. It is much more legible and clear now.

Reviewer #4: The authors have carefully addressed all of my previous comments, and the revisions have substantially improved the manuscript. I am satisfied with their responses and find no remaining issues. I therefore recommend the manuscript for acceptance in its current form.

Reviewer #5: Thank you for the opportunity to review this paper titled ‘physical activity, sleep pattern and problematic internet use in undergraduate health-science students of Nepal – a cross-sectional study’.

General comment: It is a significant and relevant contribution of public health importance, as the authors explored the patterns of computer usage, sleep habits, and physical activity among

Undergraduate health science students in Nepal, and investigated the prevalence and predictors of problematic internet use (PIU) among them. The survey revealed a relationship that exist between screen usage, PIU, physical activity and sleep among Nepal students. It therefore recommend the need for healthier lifestyle choices in the era of digital education among undergraduate students to promote physical and emotional well-being as well as enhanced academic performance among college students.

Specific comment:

Abstract: This seems adequate but the authors need to address the following concerns;

1. Line 24-26. The authors should include the percentage in the following sentence. …About a third (insert percent here ) reported inadequate sleep and a similar number were physically inactive (what percentage?), with female (insert percentage) less active than male (percentage).

2. Line 27-28, change the word ‘sex’ to ‘gender’ in the statement or be specific of implicated gender (male or female)..Multivariable analysis confirmed that sex, sleep quality…..

Introduction: This was adequate, however, there are certain unreferenced sentences here. For example;

1. Line 67-68, Nepal is also seeing a growing internet penetration rate of 51.6% in 2023, which is rising (currently 55.8% in mid-2025)..no reference

2. Line 78-83, the entire definition and description of PIU has no reference. The authors should reference this appropriately.

3. Line 92, the authors used the word studies but cited only one study at the end of the sentence. See.. Studies also underscore the correlation between periods………..(12)

Methods: The study was a cross-sectional survey and authors tried to explain the details in the methods section. However, there is need to address the following observations;

1. The authors selected a medical college in one out of 7 provinces in Nepal using purposive sampling and target population were undergraduates in MBBS, BDS, Nursing and paramedical sciences programs. What are the courses that constituted paramedical sciences in the sampled population?

2. The authors said all the consenting undergraduate students of different streams were included in the study. What did the authors meant by different streams? How did the authors achieved this? What form of sampling method was used to ensure adequate and fair representation of different streams capturing students from different programs (MBBS, Nursing BDS and paramedical sciences)?

3. What is the total population of undergraduate students involving all listed courses at the time of the study? This was not stated. This is very necessary to determine if the number sampled could be a true representation of the students studying MBBS, BDS, Nursing and Paramedical sciences.

4. The authors appeared to use convenience sampling with snow ball technique in selecting respondents which has its own draw back in this type of survey. What is the rationale for the use of snow balling, when the target population are located in the same faculty in a single college?

5. The authors used social media or phone call to reach out to potential respondents. What forms of social media handle (Whatapp, Instagram, twitter (X) etc.) was used? This needs to be specified.

6. What did authors meant by approachable students? Who are these approachable students? Are they students’ representative from each programs? How were they identify by the researchers? Please clarify

7. How did authors got the phone contacts of respondents? Please clarify

8. Instrument: The authors used semi structured questionnaire which comprised of many abridged tools to fit into the context in this study. However, it is very essential that description of these tools be detailed and organized in such a way that each adapted instrument is exhaustively described including scoring adapted for the purpose of this study before the next tool for clarity. Also, the authors shifted some discussion on the tools again under result section which may not be necessary as they ought to focus mainly on the findings or results only here. The authors captured almost all the information concerning the tools but were not described in a single and well-coordinated manner under instruments for quick understanding by the readers. For example the authors adapted questions from the Pittsburgh sleep quality index to assess sleep habits and sleep disturbances and combined the score for the two (0-17) without first describing what questions were asked under sleep habit and sleep disturbances with examples of such questions and corresponding scoring used, so as to have a clear understanding of combined scoring range of (0-17) quoted. It means the questions on sleep habits are 3 items with a scoring score 0-1, while the sleep disturbances was a 7-item tool with its own scoring (0-2). There is need to explain this clearly under a single subheading sleep assessment. I suggest the authors used this approach under instrument:

a. Demographic: describe briefly the questions that were asked under this

b. Sleep assessment: put all the tools description including examples of questions that were asked to assess sleep habits and sleep disturbances along with scoring to have a clear understanding even without going to look for your questionnaire or tables of results.

c. Physical activity: described the tool used for physical activity here and scoring method adopted under here (1PAQ).

d. Digital device: this description is adequate.

e. Problematic Internet Use (PIU) tool: described everything about this tool here, give few examples of the questions and expected responses and scoring used. Reference studies that have used the abridged version being considered in this study, if any and state if any previous validation of this tool in this population in the past in Nepal. Though authors was cleared that adapted version was review by expert and refined accordingly.

Ethics: Was there any permission sought from the college/faculty/department following the ethics approval from the Ethical Review Board of Nepal Health Research Council?

Results: This section was presented logically with a clear understanding as represented in the tables of results. However, here are few observations;

1. the information on the statistics of respondents who completed the survey from each programs (MBBS, BDS, Nursing etc) was not included as part of the demographic description of study population, which could have provided further insights on the demographic and may be subjected to further sub-analysis using outcome variables in this study. This should be acknowledged as part of the study limitation.

2. Line 191, you may rewrite the sentence by adding percentage. See… out of 358 respondents, about a third (n=109, 30.45%) got sleep less than 7 hours, and 4 (1.12%) had more than 9 hours….

3. Line 193, Nineteen (5.31%) students had prolonged sleep latency of more than 30 minutes…

4. Table 2, under sleep disturbances section, add percentage to each of the frequency by putting it in bracket after the frequency. E.g Trouble falling asleep never or rarely 159 (?%), occasionally 168 (?%)

5. The sentence on the line 197-199 (the sleep disturbances were assessed by 7 item questionnaire ……..two students did not respond to sleep disturbance questions) should be moved to instrument under sleep. Focus should be on results/findings only here not description of tool anymore

6. Line 199, the early morning waking was the most common (insert percentage for specificity and clarity) sleep disturbances. The result section is about frequency and percentage as different from discussion. The authors should take note of this.

7. There are multiple repetition of statements by the authors. For example line 201-204 where the authors were describing scoring of tools should expunged and moved appropriately to the method section under instrument. Focus should be on result interpretation only

8. Line 205, Although male sleep time was more delayed and had lower sleep duration (? time) than female…. The authors should quote supporting figure and percentage for easy understanding. This should be the approach to result writing.

9. Under physical activity, line 211-221 that is describing the physical activity tool should be moved to the method under instrument. The authors should describe the finding here. There is need to be consistent. Similarly, scoring and categorization of physical activity using IPAQ (Line 225-226) should be moved to instrument. The relevant statement should start from more than half of of the participants were minimally active (55.8%)…… male were significantly more active than females (please add relevant figure and possible p value here)

10. Line 234. The most common primary use of smartphone was scrolling some social media platform (?percent), followed by watching video and movies (?percent)……The authors should add percentage for clarity.

11. Problematic internet use (line 273-277). The statement in line 273-276 is a repetition and is not necessary under results. This should be expunged. Focus on your finding than tool description. The statement should start from the mean of the tool. Also, line 281-284 should include correlation figure or p value gotten for clarity. Any important findings worth reporting in the result section should be complete with relevant figure for understanding. While we know that readers can check others in the table presented, but certain parameter must be complete in the reporting prose.

Discussion: This was detailed enough as relevant studies were cited for comparison. Here are few observations made that need clarifications;

1. Line 324. Our study was conducted across multiple centres,….. what did authors meant by multiple centres when a single college was the study site? Please clarify

2. Line 406-409 is a repetition statement and may not be necessary. The authors can paraphrase the sentence if there is need to refer to this.

3. Limitations and future directions: This was described adequately. However, the authors should include the drawback of sampling method used in selecting respondents. This has a tendencies to attract only respondents who are interested in the subject of investigation excluding several other potential respondents that could have affected the findings of this study. The other limitation was mentioned under results section (see my observation).

Conclusion: It captured key findings, and the authors highlighted clears recommendations for further studies.

**********

7. PLOS authors have the option to publish the peer review history of their article (what does this mean?). If published, this will include your full peer review and any attached files.

**Do you want your identity to be public for this peer review?**  If you choose “no”, your identity will remain anonymous but your review may still be made public.

For information about this choice, including consent withdrawal, please see our Privacy Policy.

Reviewer #2: No

Reviewer #4: **Yes:** Seemab Mehmood

Reviewer #5: **Yes:** Dr Mumeen Olaitan Salihu

**********

[NOTE: If reviewer comments were submitted as an attachment file, they will be attached to this email and accessible via the submission site. Please log into your account, locate the manuscript record, and check for the action link "View Attachments". If this link does not appear, there are no attachment files.] **Figure resubmission:**   While revising your submission, we strongly recommend that you use PLOS’s NAAS tool (https://ngplosjournals.pagemajik.ai/artanalysis) to test your figure files. NAAS can convert your figure files to the TIFF file type and meet basic requirements (such as print size, resolution), or provide you with a report on issues that do not meet our requirements and that NAAS cannot fix.

After uploading your figures to PLOS’s NAAS tool - https://ngplosjournals.pagemajik.ai/artanalysis, NAAS will process the files provided and display the results in the "Uploaded Files" section of the page as the processing is complete. If the uploaded figures meet our requirements (or NAAS is able to fix the files to meet our requirements), the figure will be marked as "fixed" above. If NAAS is unable to fix the files, a red "failed" label will appear above. When NAAS has confirmed that the figure files meet our requirements, please download the file via the download option, and include these NAAS processed figure files when submitting your revised manuscript. **Reproducibility:**  To enhance the reproducibility of your results, we recommend that authors of applicable studies deposit laboratory protocols in protocols.io, where a protocol can be assigned its own identifier (DOI) such that it can be cited independently in the future. Additionally, PLOS ONE offers an option to publish peer-reviewed clinical study protocols. Read more information on sharing protocols at https://plos.org/protocols?utm_medium=editorial-email&utm_source=authorletters&utm_campaign=protocols

Attachment Submitted filename: REVIEWER COMMENTS-Plos Digital Health-09.11.25.docx

# Author comment: Author response to Decision Letter 3

**DOI:** 10.1371/journal.pdig.0000797.r006

3 Jan 2026

Attachment Submitted filename: Response to Reviewers 3.doc

# Peer review: Decision Letter 3

**DOI:** 10.1371/journal.pdig.0000797.r007

**Authors:** Dukyong Yoon, Margaret Isioma Ojeahere

18 Mar 2026

PDIG-D-25-00111R3­Physical activity, sleep pattern and problematic internet use in undergraduate health-science students of Nepal – a cross-sectional studyPLOS Digital Health Dear Dr. Jha,Thank you for submitting your manuscript to PLOS Digital Health. After careful consideration, we feel that it has merit but does not fully meet PLOS Digital Health's publication criteria as it currently stands. Therefore, we invite you to submit a revised version of the manuscript that addresses the points raised during the review process.Please submit your revised manuscript by Apr 17 2026 11:59PM. If you will need more time than this to complete your revisions, please reply to this message or contact the journal office at digitalhealth@plos.org. When you're ready to submit your revision, log on to https://www.editorialmanager.com/pdig/ and select the 'Submissions Needing Revision' folder to locate your manuscript file. Please include the following items when submitting your revised manuscript:* A letter that responds to each point raised by the editor and reviewer(s). You should upload this letter as a separate file labeled 'Response to Reviewers'. This file does not need to include responses to any formatting updates and technical items listed in the 'Journal Requirements' section below.* A marked-up copy of your manuscript that highlights changes made to the original version. You should upload this as a separate file labeled 'Revised Manuscript with Track Changes'.* An unmarked version of your revised paper without tracked changes. You should upload this as a separate file labeled 'Manuscript'.If you would like to make changes to your financial disclosure, competing interests statement, or data availability statement, please make these updates within the submission form at the time of resubmission. Guidelines for resubmitting your figure files are available below the reviewer comments at the end of this letter.We look forward to receiving your revised manuscript. Kind regards,Margaret Isioma Ojeahere, M.D., MSc., FWACPAcademic EditorPLOS Digital HealthMargaret OjeahereAcademic EditorPLOS Digital HealthLeo Anthony CeliEditor-in-ChiefPLOS Digital Health orcid.org/0000-0001-6712-6626**Journal Requirements:** If the reviewer comments include a recommendation to cite specific previously published works, please review and evaluate these publications to determine whether they are relevant and should be cited. There is no requirement to cite these works unless the editor has indicated otherwise.  **Additional Editor Comments (if provided):** [Note: HTML markup is below. Please do not edit.]**Reviewers' Comments:** Reviewer's Responses to Questions

**Comments to the Author**

1. If the authors have adequately addressed your comments raised in a previous round of review and you feel that this manuscript is now acceptable for publication, you may indicate that here to bypass the “Comments to the Author” section, enter your conflict of interest statement in the “Confidential to Editor” section, and submit your "Accept" recommendation.

Reviewer #4: All comments have been addressed

Reviewer #5: All comments have been addressed

**********

2. Does this manuscript meet PLOS Digital Health’s publication criteria? Is the manuscript technically sound, and do the data support the conclusions? The manuscript must describe methodologically and ethically rigorous research with conclusions that are appropriately drawn based on the data presented.

Reviewer #4: Yes

Reviewer #5: Yes

**********

3. Has the statistical analysis been performed appropriately and rigorously?

Reviewer #4: Yes

Reviewer #5: Yes

**********

4. Have the authors made all data underlying the findings in their manuscript fully available (please refer to the Data Availability Statement at the start of the manuscript PDF file)?

The PLOS Data policy requires authors to make all data underlying the findings described in their manuscript fully available without restriction, with rare exception. The data should be provided as part of the manuscript or its supporting information, or deposited to a public repository. For example, in addition to summary statistics, the data points behind means, medians and variance measures should be available. If there are restrictions on publicly sharing data—e.g. participant privacy or use of data from a third party—those must be specified.

Reviewer #4: Yes

Reviewer #5: Yes

**********

5. Is the manuscript presented in an intelligible fashion and written in standard English?

PLOS Digital Health does not copyedit accepted manuscripts, so the language in submitted articles must be clear, correct, and unambiguous. Any typographical or grammatical errors should be corrected at revision, so please note any specific errors here.

Reviewer #4: No

Reviewer #5: Yes

**********

6. Review Comments to the Author

Please use the space provided to explain your answers to the questions above. You may also include additional comments for the author, including concerns about dual publication, research ethics, or publication ethics. (Please upload your review as an attachment if it exceeds 20,000 characters)

Reviewer #4: Thank you for your valuable work and for submitting the revised manuscript. There remain a few instances where sentence structure could be further refined to improve clarity.

Lines 22, 103, 108, 114, 119, and throughout the manuscript: Consider replacing “undergraduate health-science students” with “undergraduate health-sciences students.”

Lines 88–90: Consider revising

“…showed that Nepal had the highest prevalence of PIU as per the Generalized Problematic Internet Use Scale-2 (GPIUS2) scale (12.6%) (9).”

to

“…showed that Nepal had the highest prevalence of PIU (12.6%) as per the Generalized Problematic Internet Use Scale-2 (GPIUS2) (9).”

Reviewer #5: Thank you for the opportunities to review the revised manuscript. All observations made at the last round of review have been addressed satisfactorily by the authors.

**********

7. PLOS authors have the option to publish the peer review history of their article (what does this mean?). If published, this will include your full peer review and any attached files.

**Do you want your identity to be public for this peer review?**  If you choose “no”, your identity will remain anonymous but your review may still be made public.

For information about this choice, including consent withdrawal, please see our Privacy Policy.

Reviewer #4: **Yes:** Seemab Mehmood

Reviewer #5: **Yes:** Mumeen Olaitan Salihu

**********

[NOTE: If reviewer comments were submitted as an attachment file, they will be attached to this email and accessible via the submission site. Please log into your account, locate the manuscript record, and check for the action link "View Attachments". If this link does not appear, there are no attachment files.] **Figure resubmission:**   While revising your submission, we strongly recommend that you use PLOS’s NAAS tool (https://ngplosjournals.pagemajik.ai/artanalysis) to test your figure files. NAAS can convert your figure files to the TIFF file type and meet basic requirements (such as print size, resolution), or provide you with a report on issues that do not meet our requirements and that NAAS cannot fix.

After uploading your figures to PLOS’s NAAS tool - https://ngplosjournals.pagemajik.ai/artanalysis, NAAS will process the files provided and display the results in the "Uploaded Files" section of the page as the processing is complete. If the uploaded figures meet our requirements (or NAAS is able to fix the files to meet our requirements), the figure will be marked as "fixed" above. If NAAS is unable to fix the files, a red "failed" label will appear above. When NAAS has confirmed that the figure files meet our requirements, please download the file via the download option, and include these NAAS processed figure files when submitting your revised manuscript. **Reproducibility:**  To enhance the reproducibility of your results, we recommend that authors of applicable studies deposit laboratory protocols in protocols.io, where a protocol can be assigned its own identifier (DOI) such that it can be cited independently in the future. Additionally, PLOS ONE offers an option to publish peer-reviewed clinical study protocols. Read more information on sharing protocols at https://plos.org/protocols?utm_medium=editorial-email&utm_source=authorletters&utm_campaign=protocols

# Author comment: Author response to Decision Letter 4

**DOI:** 10.1371/journal.pdig.0000797.r008

8 Apr 2026

Attachment Submitted filename: Response to Reviewers 4.doc

# Peer review: Decision Letter 4

**DOI:** 10.1371/journal.pdig.0000797.r009

**Authors:** Dukyong Yoon, Margaret Isioma Ojeahere

29 May 2026

­Physical activity, sleep pattern and problematic internet use in undergraduate health-sciences students of Nepal – a cross-sectional study

PDIG-D-25-00111R4

Dear Dr Jha,

We are pleased to inform you that your manuscript '­Physical activity, sleep pattern and problematic internet use in undergraduate health-sciences students of Nepal – a cross-sectional study' has been provisionally accepted for publication in PLOS Digital Health.

Before your manuscript can be formally accepted you will need to complete some formatting changes, which you will receive in a follow-up email from a member of our team.

Please note that your manuscript will not be scheduled for publication until you have made the required changes, so a swift response is appreciated.

IMPORTANT: The editorial review process is now complete. PLOS will only permit corrections to spelling, formatting or significant scientific errors from this point onwards. Requests for major changes, or any which affect the scientific understanding of your work, will cause delays to the publication date of your manuscript.

If your institution or institutions have a press office, please notify them about your upcoming paper to help maximize its impact. If they'll be preparing press materials, please inform our press team as soon as possible -- no later than 48 hours after receiving the formal acceptance. Your manuscript will remain under strict press embargo until 2 pm Eastern Time on the date of publication. For more information, please contact digitalhealth@plos.org.

Thank you again for supporting Open Access publishing; we are looking forward to publishing your work in PLOS Digital Health.

Best regards,

Margaret Isioma Ojeahere, M.D., MSc., FWACP

Academic Editor

PLOS Digital Health

***********************************************************

**Additional Editor Comments (if provided):**

**Reviewer Comments (if any, and for reference):**

Reviewer's Responses to Questions

**Comments to the Author**

1. If the authors have adequately addressed your comments raised in a previous round of review and you feel that this manuscript is now acceptable for publication, you may indicate that here to bypass the “Comments to the Author” section, enter your conflict of interest statement in the “Confidential to Editor” section, and submit your "Accept" recommendation.

Reviewer #4: All comments have been addressed

**********

2. Does this manuscript meet PLOS Digital Health’s publication criteria? Is the manuscript technically sound, and do the data support the conclusions? The manuscript must describe methodologically and ethically rigorous research with conclusions that are appropriately drawn based on the data presented.

Reviewer #4: Yes

**********

3. Has the statistical analysis been performed appropriately and rigorously?

Reviewer #4: Yes

**********

4. Have the authors made all data underlying the findings in their manuscript fully available (please refer to the Data Availability Statement at the start of the manuscript PDF file)?

The PLOS Data policy requires authors to make all data underlying the findings described in their manuscript fully available without restriction, with rare exception. The data should be provided as part of the manuscript or its supporting information, or deposited to a public repository. For example, in addition to summary statistics, the data points behind means, medians and variance measures should be available. If there are restrictions on publicly sharing data—e.g. participant privacy or use of data from a third party—those must be specified.

Reviewer #4: Yes

**********

5. Is the manuscript presented in an intelligible fashion and written in standard English?

PLOS Digital Health does not copyedit accepted manuscripts, so the language in submitted articles must be clear, correct, and unambiguous. Any typographical or grammatical errors should be corrected at revision, so please note any specific errors here.

Reviewer #4: Yes

**********

6. Review Comments to the Author

Please use the space provided to explain your answers to the questions above. You may also include additional comments for the author, including concerns about dual publication, research ethics, or publication ethics. (Please upload your review as an attachment if it exceeds 20,000 characters)

Reviewer #4: Thank you so much for revising the manuscript. It seems to become a good read!

**********

7. PLOS authors have the option to publish the peer review history of their article (what does this mean?). If published, this will include your full peer review and any attached files.

**Do you want your identity to be public for this peer review?**  If you choose “no”, your identity will remain anonymous but your review may still be made public.

For information about this choice, including consent withdrawal, please see our Privacy Policy.

Reviewer #4: **Yes:** Seemab Mehmood

**********
